# Integrated metabolomic profiling reveals metabolomic responses by epicardial and myocardial stromal cells to ischemia

**DOI:** 10.1007/s11306-026-02438-0

**Published:** 2026-04-29

**Authors:** Dongwei Sun, Alex Postajian, Edgmin Rostomian, Yu Chen, Junyoung O. Park, Vedi Hatamian, Kevin Babakhan Vartanian, Finosh G. Thankam

**Affiliations:** 1 Smidt Heart Institute, Cedars-Sinai Medical Center, Los Angeles, CA 90048 USA; 2 Department of Translational Research, College of Osteopathic Medicine of the Pacific, Western University of Health Sciences, 309 E. Second Street, Pomona, CA 91766-1854 USA; 3 Molecular Instrumentation Center, University of California-Los Angeles, Los Angeles, CA 90095 USA; 4 Department of Chemical and Biomolecular Engineering, University of California, Los Angeles, Los Angeles, CA 90095 USA

**Keywords:** Myocardial ischemia, Metabolic reprogramming, Epicardial adipose stromal cells, Ventricular stromal cells, Cardiac homeostasis

## Abstract

**Introduction:**

Cardiac ischemia induces substantial metabolomic reprogramming, which dysregulates cardiomyocytes (CMs) and non-myocyte stromal cell populations. The stromal cells derived from epicardial adipose tissue (EAT) and ventricle are critical for extracellular matrix (ECM) remodeling, paracrine signaling, and myocardial homeostasis. However, the metabolomic content and responses of EAT-derived stromal cells (EATDS) and ventricular stromal cells (VSCs) remain unknown.

**Methodology:**

This study employed untargeted liquid chromatography–mass spectrometry (LC-MS)-based metabolomics to characterize ischemia-driven metabolic reprogramming in EATDS and VSCs harvested from swine hearts. Ischemia was simulated using the standard ischemic buffer (pH 6.2) for 2 h.

**Results:**

Metabolomic screening revealed 65 and 68 metabolites, respectively, for EATDS and VSCs. Results revealed extensive downregulation of amino acid biosynthesis, redox pathways, and mitochondrial metabolism, alongside selective upregulation of glycolytic and cofactor-associated metabolites. Pathway enrichment analyses indicated significant suppression of the TCA cycle, one-carbon metabolism, glutathione cycling, and branched-chain amino acid degradation, reflecting impaired bioenergetic and antioxidant capacity. Adaptive responses included the enrichment of glycolysis, β-alanine, and glyoxylate/dicarboxylate metabolism, consistent with metabolic plasticity under hypoxic conditions. Network-based analyses linked these metabolic shifts to inflammatory pathways. Functional assays demonstrated that sarcosine, pyroglutamic acid, and 3-hydroxypropionic acid modulate the gene expression of cardiac regenerative biomarkers, including GATA4, Nkx2.5, TROP-I, LGALS1, TBX5, and IRX4.

**Conclusions:**

These findings suggest that ischemia-induced metabolomic changes exert transcriptional control over cardiac remodeling programs, emphasizing the regulatory potential of metabolite-gene interactions. Such an integrated metabolomic transcriptional response highlights novel therapeutic targets for modulating cellular resilience and heart regeneration following ischemic heart disease.

**Supplementary Information:**

The online version contains supplementary material available at 10.1007/s11306-026-02438-0.

## Introduction

Myocardial ischemia is marked by insufficient oxygen delivery to the heart tissue, mostly as a result of coronary artery blockage, which sets off a series of metabolic and molecular abnormalities in the cardiac cells (Angelico et al., 2023; Sant’Anna-Silva et al., 2021). The pathophysiology of ischemia extends beyond cardiomyocytes (CMs), encompassing the broader cardiac microenvironment, including epicardial adipose tissue (EAT) and ventricular stromal cells (VSCs), which collectively contribute to myocardial homeostasis and remodeling under stress conditions (Martínez-Reyes & Chandel, 2020; Zeng et al., 2025). EAT, a metabolically active visceral fat depot, is a repository of functionally active stromal cells. EAT-derived stromal cells (EATDS) intimately interact with the myocardium through paracrine and vasocrine signaling that modulates local inflammation, oxidative stress, and energetics (Albaugh et al., 2017; Karsdal et al., 2025). Similarly, we have reported that VSCs, the predominant non-myocyte population in the heart, are central to extracellular matrix (ECM) regulation, cardiac healing, and tissue remodeling in response to ischemic injury (Thankam, La, et al. 2023; Thai et al., 2025; Cha et al., 2024).

Ischemia creates a hypoxic environment that disrupts mitochondrial oxidative phosphorylation, resulting in a rapid metabolic shift toward anaerobic glycolysis, downregulation of biosynthetic pathways, and redox imbalance (Lesnefsky et al. 2017). Interestingly, our previous research established the cardioprotective communication between EATDS and VSCs, in responding to ischemia, via secretory vesicles (Thankam, Sedighim, et al. 2023). While the metabolic adaptations of CMs to ischemia have been extensively studied, the specific reprogramming events occurring in EATDS and VSCs under ischemic stress remain underexplored. Hence, understanding these alterations is essential, as both cell types exhibit dynamic metabolic phenotypes and actively influence cardiac repair.

Prior research has demonstrated that ischemia induces metabolic alterations in cardiac metabolomics (Winyard et al., 2005; Ducker & Rabinowitz, 2017; Berridge, 2016). Since EATDS and VSCs reflect the implications of cardiac ischemia, we propose that the metabolomic patterning/alteration in these cells would reveal their protective adaptations to withstand the deleterious effects of ischemia. However, a comprehensive metabolomic profiling of EATDS and VSCs under ischemic stress integrated with systems-level network tracking remains lacking. Additionally, the contribution of metabolite-driven signaling to transcriptional regulation in cardiac repair contexts has not been thoroughly investigated.

**Fig. 1:**
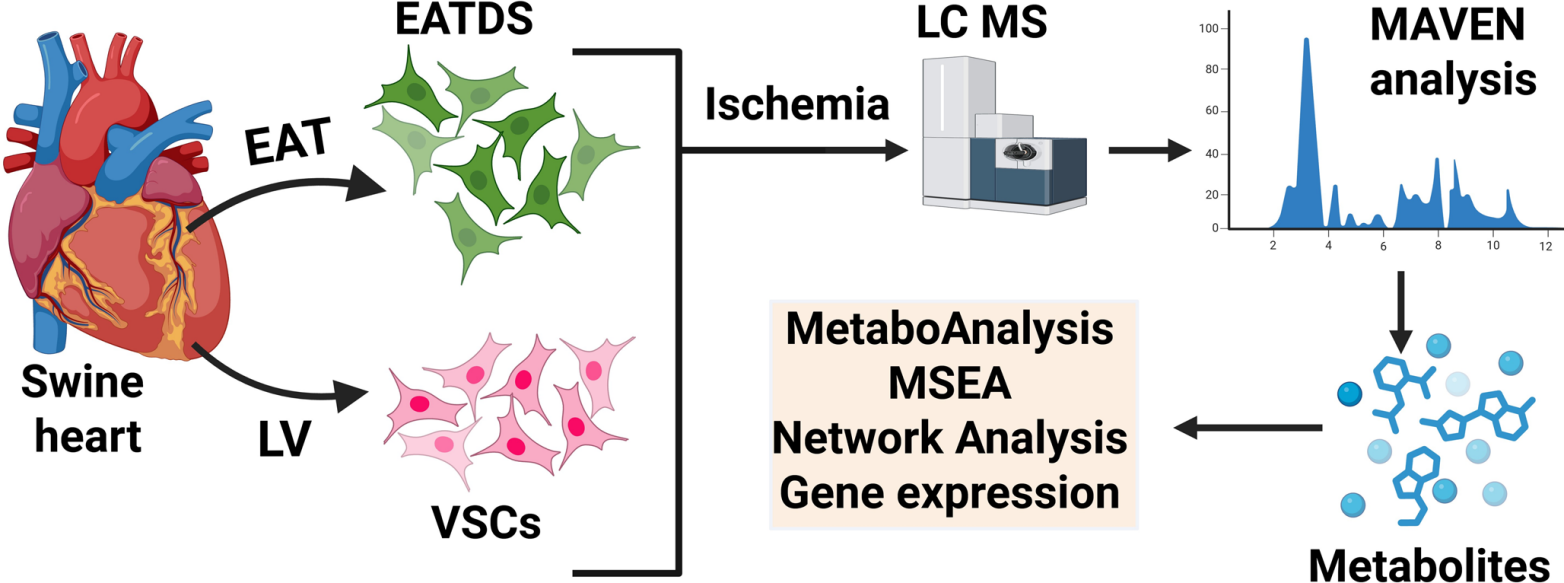
Schematic representation of the overall approach and workflow of the study. Briefly, the EATDS and VSCs were harvested from the EAT (epicardial adipose tissue) and LV (left ventricle) respectively, challenged under ischemia, performed metabolite screening using LC-MS and the interconnecting mediators and pathways were determined using MSEA and network analysis. The EATDS and VSCs were treated with highly regulated mediators to assess the cardiac regenerative genes

The hypothesis of our study was that ischemia induces distinct and functionally relevant metabolomic reprogramming in epicardial adipose tissue-derived stromal cells (EATDS) and ventricular stromal cells (VSCs), which contribute to the regulation of transcriptional programs involved in cardiac repair (Fig. 1).Our objectives are to comprehensively characterize ischemia-induced metabolomic alterations in EATDS and VSCs using LC-MS-based metabolomics, to evaluate the functional impact of selected metabolites on the transcriptional regulation of cardiac repair-related genes, thereby providing insight into metabolite-associated pathways that may contribute to stromal cell-mediated cardiac remodeling under ischemic conditions.

## Materials and methods

### Isolation, culture, and maintenance of EATDS and VSCs

EATDS and VSCs were isolated respectively from the EAT and myocardium of Yucatan miniswine (*Sus scrofa*, Sinclair BioResources) (*n* = 3, female) following a collagenase digestion technique, as per our previously validated protocols (Thankam, La, et al. 2023) (Thankam and Agrawal 2022). All procedures involving animals were reviewed and approved by the Institutional Animal Care and Use Committee (IACUC) at Western University of Health Sciences, Pomona, California. Prior to tissue collection, the animals were humanely sacrificed strictly following IACUC guidelines. The harvested cells were cultured under sterile conditions using Dulbecco’s Modified Eagle Medium (DMEM, high glucose) supplemented with 10% fetal bovine serum (FBS) for EATDS and 20% FBS for VSCs, maintained at 37 °C in a humidified incubator with 5% CO₂ and standard antibiotic supplementation. Anatomical consistency was ensured by collecting tissue samples from the same tissue locations across all the animals. The isolation and characterization of EATDS and VSCs were detailed in our previous publications (Thankam, Sedighim, et al. 2023; Thankam and Agrawal 2022). EATDS and VSCs of passages 0–4 were used for all experiments. Ischemia was simulated by challenging EATDS and VSCs using a defined ischemic buffer (containing 118 mM NaCl, 24 mM Na₂HCO₃, 1 mM Na₂HPO₄, 2.5 mM CaCl₂, 1.2 mM MgCl₂, 20 mM sodium lactate, 16 mM KCl, and 10 mM 2-deoxyglucose, adjusted to pH 6.2) for 2 h (Thankam, Sedighim, et al. 2023; Thankam and Agrawal 2022). Control cells were maintained under standard culture conditions in complete DMEM without ischemia challenge.

### Metabolomic profiling and differential metabolite analysis using liquid chromatography-mass spectrometry (LC-MS)

EATDS and VSC samples (*n* = 4) were processed following standard metabolite extraction protocols and analyzed using LC-MS to generate raw spectral data, as we reported (Lakhani et al. 2024). Extracts were analyzed on a Vanquish Duo UHPLC system coupled to a Q Exactive Plus orbitrap mass spectrometer (ThermoFisher) by electrospray ionization. The LC separation was performed on a XBridge BEH Amide XP column (150 mm × 2.1 mm, 2.5 μm particle size, Waters, Milford, MA) using a gradient of solvent A (95:5 water/acetonitrile with 20 mM ammonium acetate, 20 mM ammonium hydroxide, pH 9.4) and solvent B (acetonitrile with 1% H_2_O). The gradient was 0 min, 90% B; 2 min, 90% B; 3 min, 75% B; 7 min, 75% B; 8 min, 70% B; 9 min, 70% B; 10 min, 50% B; 12 min, 50% B; 13 min, 25% B; 14 min, 25% B; 16 min, 0% B; 20 min, 0% B; 23 min, 0% B; 27 min, 25% B; 30 min, 45% B; 33 min, 90% B; 40 min, 90% B. The flow rate was 150 µL min^− 1^. Injection volume was 5 µL, and autosampler and column temperatures were 4 °C and 25 °C, respectively. The MS operated in negative and positive ion modes with a resolution of 140,000 at mass-to-charge ratio (m/z) 200 with scan range of m/z 60–2000. With retention times determined by authenticated standards, resulting mass spectra and chromatograms were identified and integrated using the Metabolomic Analysis and Visualization Engine (MAVEN) (Seitzer et al. 2022). Metabolites were annotated in accordance with the Metabolomics Standards Initiative (MSI) guidelines.

Peaks corresponding to individual metabolites were carefully curated, and peak intensities were extracted across all samples to construct the metabolite intensity matrix. Following data preprocessing, normalization was applied to the intensity values to account for technical variability. Data counts from the ischemic cells were normalized with that of average from the corresponding control. The normalized data were converted into Log2 fold change where the control values were transformed to zero while positive values signify upregulation and negative values denote downregulation. Average log₂-transformed intensities (Log_2_ fold change) were calculated for each metabolite within experimental groups to facilitate comparison. To highlight key metabolic alterations, metabolites with the largest positive and negative fold changes were selected for detailed discussion.

### Pathway and network-based metabolomic analysis

For comprehensive pathway-level interpretation, the full set of detected metabolites and their corresponding FC were subjected to downstream analysis using MetaboAnalyst 6.0 (www.metaboanalyst.ca). Uploaded data were analyzed through multiple modules within the platform, including pathway enrichment analysis, metabolite set enrichment analysis (MSEA), and network analysis. Annotation of metabolites was performed using the Kyoto Encyclopedia of Genes and Genomes (KEGG) database to systematically map altered metabolites to known biological pathways, molecular functions, and disease processes. These analyses provided insights into the systemic metabolic dysregulation associated with alterations in amino acid metabolism, mitochondrial function, redox homeostasis, and extracellular matrix remodeling.

### Quantitative real-time PCR (qRT-PCR)

To investigate the transcriptional consequences of ischemia-induced metabolic reprogramming, three metabolites, sarcosine, (R)(+) 2-pyrrolidone-5-carboxylic acid (pyroglutamic acid), and 3-hydroxypropionic acid were selected for further functional evaluation based on their significant alterations under ischemic conditions and their involvement in key metabolic pathways relevant to cardiac homeostasis. EATDS and VSCs were treated with a sub-optimal concentration of 25 µg/ml, and the expression level of CM-specific genes such as GATA4, NKX2.5, TROP-I, LGALS1, TBX5, and IRX4 were quantified. Total RNA was extracted from control and ischemia-challenged EATDS and VSCs following the TRIol method, following the manufacturer’s protocol. For each sample, 500 ng of total RNA was used to synthesize complementary DNA (cDNA) in a 20 µL reaction volume using the cDNA synthesis kit (AZ-1996, Azura Genomics Inc., Raynham, MA, USA), according to the supplier’s instructions. Quantitative PCR amplification of GATA4, NKX2.5, TROP-I, LGALS1, TBX5, and IRX4 was performed using a C1000™ Thermal Cycler (Bio-Rad Laboratories, Hercules, CA, USA) employing SYBR Green chemistry. Each gene was amplified using specific forward and reverse primers (Table 1). The PCR conditions were denaturation at 95 °C for 10 min, followed by 40 cycles of 95 °C for 15 s and 60 °C for 1 min. The expression levels of the target genes were normalized to 18 S rRNA, which served as the internal control. All reactions were carried out in biological triplicate. Relative mRNA expression was calculated and expressed as Log2 FC relative to the corresponding control group.

**Table 1:** qPCR primer sequences used for gene expression analysis

Gene	Forward primer	Reverse primer
GATA4	5′-TGC AAT GCG GAA AGA GGG GA-3′	5′-ATC TCT TCG CTG CTG CTG GT-3′
NKX2.5	5′-CCT TCT ACC CGC GTG CCT-3′	5′-CGT AGA CCT GTG CCT GCG AA-3′
TROP-I	5′-GAG CCG CAC GCC AAG AAA AA-3′	5′-CGC AAT CTG CAG CAT CAG GG-3′
LGALS1	5′-CAG CCT GGA AGT GTC GTG GA-3′	5′-AGG CGG TTG GGG AAA CTG AA-3′
TBX5	5′-AGC TCG GCG AAG GGA TGT AT-3′	5′-AGA GAA ACT CTG GGG GCT GG-3′
IRX4	5′-TAC CCC TAC TCC TCC GCA CC-3′	5′-GTA GAC CGG GCA GTA GAC GG-3′
18 s rRNA	5′-ACG TTG GCG AGA GCG TGG-3′	5′-AGG TGG AGG AGG CGA GAG AG-3′

### Statistical analysis

The mass spectrometry and PCR results were expressed as mean ± standard deviation (SD). All statistical analyses were performed using GraphPad Prism software (version 9). Statistical evaluation was performed using a one-way analysis of variance (ANOVA) followed by Dunnett’s post hoc test to compare each experimental group against the control. Experiments were independently replicated in triplicate or quadruplicate, and differences were considered statistically significant at *P* < 0.05. In addition, metabolites were considered significantly altered based on a combination of fold-change and statistical significance.

## Results

EATDS and VSCs are metabolically active cellular components of the heart that contribute significantly to myocardial homeostasis. During ischemia, these cell types are exposed to pathological stress that perturbs their metabolic equilibrium. This study employed LC-MS to characterize metabolomic alterations in cultured EATDS and VSCs under ischemia challenge (Fig. 1). Compared to control conditions, the analysis revealed extensive downregulation of critical metabolic intermediates and selective upregulation of stress-adaptive metabolites in the ischemic cells, implicating systemic dysregulation across amino acid, energy, redox, and mitochondrial pathways. Comprehensive pathway enrichment and network analyses further elucidated the molecular pathways underlying this metabolic shift.

### LC-MS profiling revealed global metabolic alterations in ischemic EATDS

**Fig. 2:**
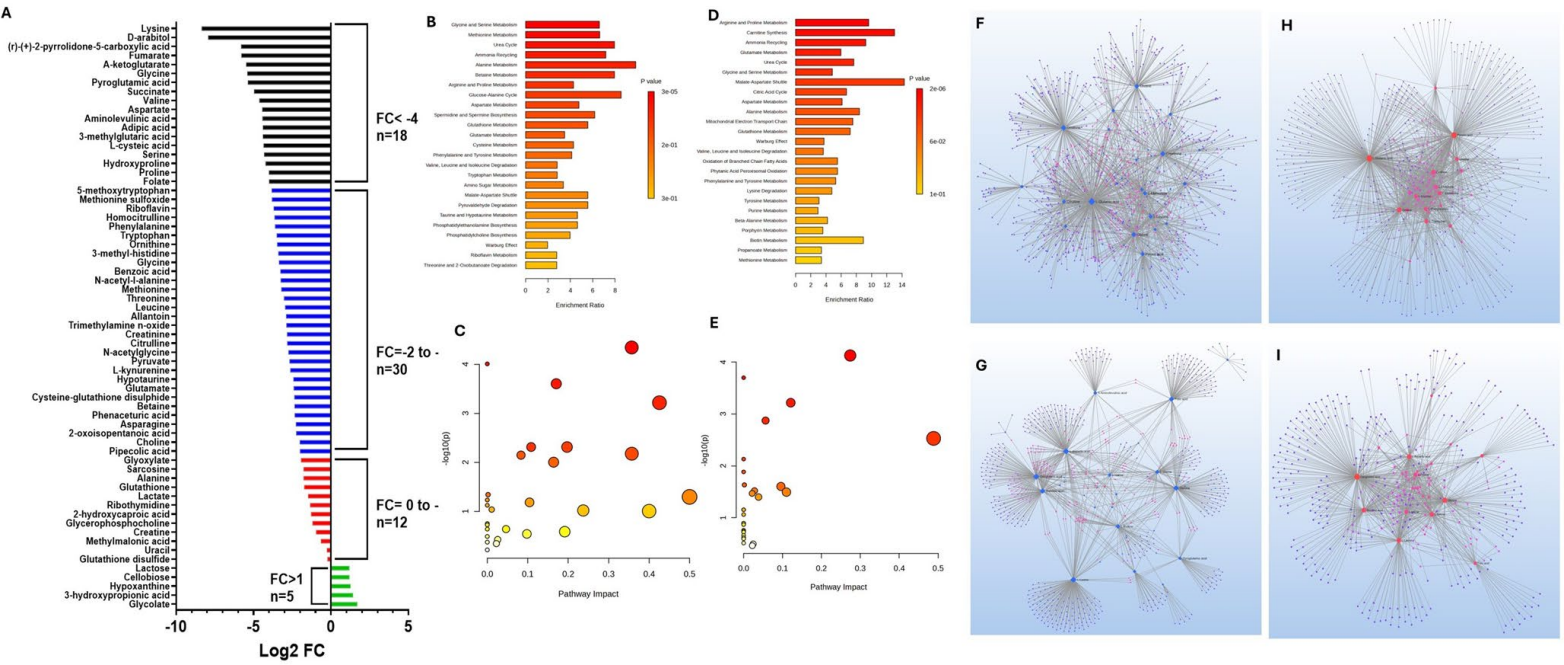
**A** Bar diagram showing 65 metabolites detected in the ischemic EATDS based on the Log2 FC. **B** MetaboAnalyst 6.0 pathway enrichment analysis of metabolites significantly downregulated (FC − 2 to − 4) in ischemia challenged EATDS (*p* < 0.05). **C** MSEA using MetaboAnalyst 6.0 showing significantly impacted pathways on -log10(p) associated with the metabolites (FC − 2 to − 4) (data in Table S3a). **D** MetaboAnalyst 6.0 pathway enrichment analysis of metabolites significantly downregulated (FC<−4) in ischemia challenged EATDS (*p* < 0.05). **E** MSEA using MetaboAnalyst 6.0 showing significantly impacted pathways on -log10(p) associated with the metabolites (FC < 4) (data in Table S3b). Metabolite-gene-disease interaction network associated with **F** moderately downregulated and **G** highly downregulated metabolites in EATDS. Metabolite-metabolite interaction network associated with **H** moderately downregulated and **I** highly downregulated metabolites in EATDS

A total of 65 metabolites were detected, among which 5 were upregulated and 48 were significantly downregulated in ischemia-challenged EATDS (Fig. 2A). Downregulated metabolites were categorized into moderately downregulated (*n* = 30, FC: −2.06 to − 4.00) and severely downregulated (*n* = 18, FC: −4.00 to − 8.40) groups (Table S1). The most profoundly affected pathways associated with these metabolites were amino acid metabolism, glutathione metabolism, one-carbon metabolism, and the tricarboxylic acid (TCA) cycle, where amino acid metabolism exhibited the most extensive downregulation.

Glutathione and redox metabolism were markedly reduced. Key intermediates such as glycine (FC = − 5.46), pyroglutamic acid (FC = − 5.42), cysteine-glutathione disulfide (FC = − 2.42), and cysteic acid (FC = − 4.41) were decreased, indicating impaired antioxidant defense and thiol homeostasis. In the one-carbon metabolism axis, folate (FC = − 4.05) and methionine (FC = − 3.26) were reduced, suggesting compromised methylation capacity and nucleotide biosynthesis.

Additionally, methyl donors and osmolytes, including betaine (FC = − 2.41), choline (FC = − 2.07), and trimethylamine N-oxide (FC = − 2.93), were decreased. The TCA cycle was strongly suppressed, with succinate (FC = − 5.02), α-ketoglutarate (FC = − 5.53), and fumarate (FC = − 5.84) markedly reduced, indicating mitochondrial dysfunction. The most pronounced decreases were observed in lysine (FC = − 8.40) and D-arabitol (FC = − 7.98) (Table S1a).

In contrast, a small subset of metabolites was upregulated, suggesting a limited adaptive response. Glycolate (FC = 1.75) showed the highest increase, followed by 3-hydroxypropionic acid (FC = 1.47) and hypoxanthine (FC = 1.31). Modest increases in cellobiose (FC = 1.24) and lactose (FC = 1.24) were also observed, indicating potential shifts in carbohydrate metabolism (Table S1a).

### Pathway enrichment analysis revealed suppression of core metabolic pathways and limited adaptive remodeling in ischemic EATDS

Pathway enrichment analysis revealed extensive disruption of amino acid, redox, and mitochondrial metabolic pathways (Table S2a–c). Among moderately downregulated metabolites (Fig. 2B), glycine, serine, and threonine metabolism were the most enriched pathways, followed by valine, leucine, and isoleucine biosynthesis and arginine biosynthesis. Additional enrichment was observed in glutathione metabolism and alanine, aspartate, and glutamate metabolism, indicating impairment in amino acid interconversion, nitrogen handling, and antioxidant capacity. The analysis of highly downregulated metabolites further highlighted alanine, aspartate, and glutamate metabolism, arginine biosynthesis, and the TCA cycle, indicating more pronounced disruption of mitochondrial function and amino acid cycling (Fig. 2D), Metabolite set enrichment analysis (MSEA) further expanded the range of affected pathways. Moderately enriched pathways included phenylalanine and tyrosine metabolism, valine, leucine, and isoleucine degradation, malate–aspartate shuttle, and the Warburg effect, indicating broader perturbations in energy metabolism and redox balance (Fig. 2C; Table S2a).

In addition to glycine and serine metabolism and methionine metabolism, strongly enriched pathways included the urea cycle and ammonia recycling, suggesting impaired nitrogen disposal. Additional pathways such as arginine and proline metabolism, betaine metabolism, glucose–alanine cycle, and glutathione-related pathways were also enriched (Fig. 2E, Table S2b).

**Fig. 3:**
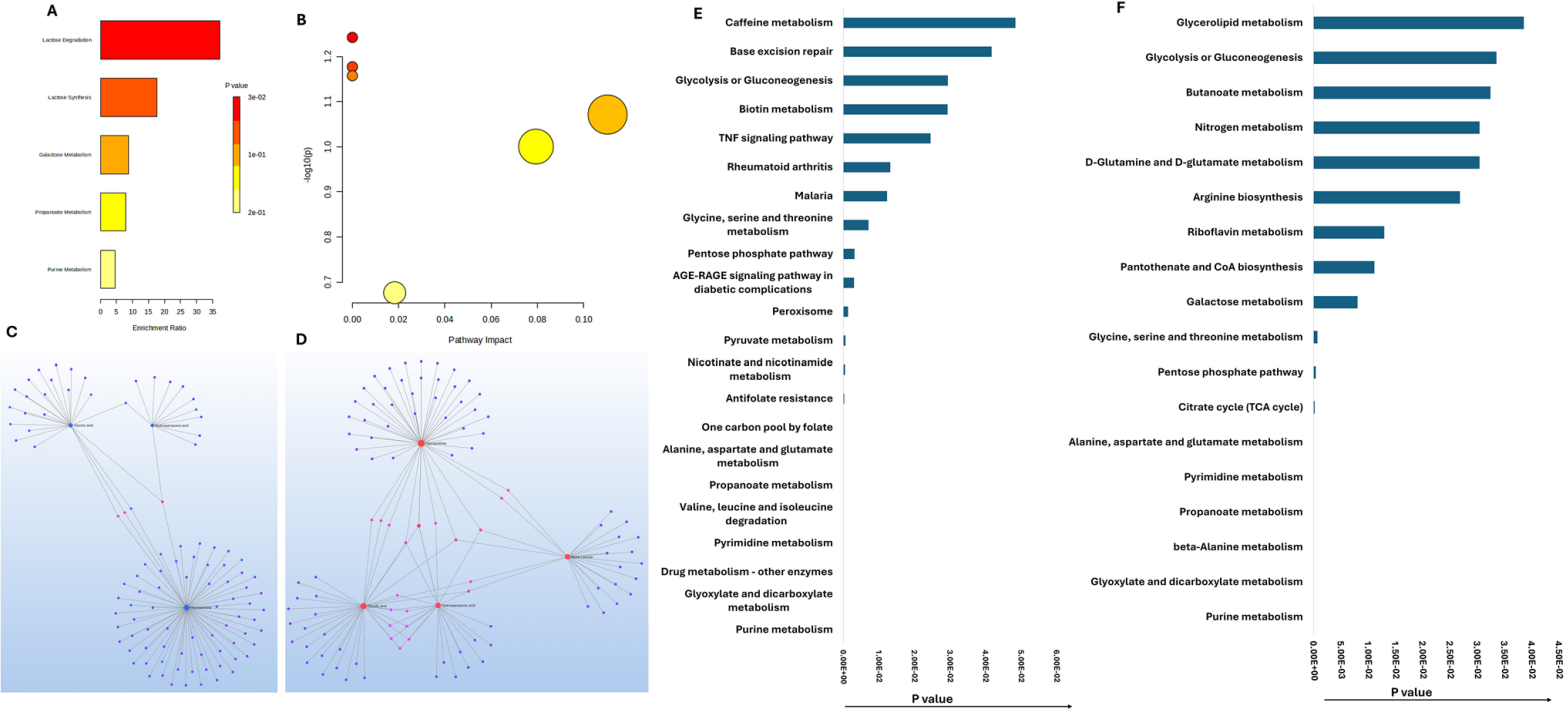
**A** MetaboAnalyst 6.0 pathway enrichment analysis of metabolites significantly upregulated in ischemia challenged EATDS (*p* < 0.05). **B** MSEA using MetaboAnalyst 6.0 showing significantly impacted pathways on -log10(p) associated with the upregulated metabolites in EATDS (data in Table S3c). **C** Metabolite-gene-disease interaction network and **D **metabolite-metabolite interaction network associated with upregulated metabolites in EATDS. The pathways enriched based on **E** metabolite-gene-disease interaction network and **F** metabolite-metabolite interaction network

Enrichment analysis of upregulated metabolites revealed modest alterations in several carbohydrate-related pathways (Fig. 3A and B; Table S2c). These findings suggest a shift in carbohydrate utilization and engagement of auxiliary metabolic routes. However, these enrichments did not reach statistical significance after multiple testing correction, indicating that these changes likely reflect limited and compensatory metabolic responses rather than robust pathway activation.

### Network-level analyses reveal integrated metabolic and signaling disruptions in ischemic EATDS

Network-based metabolite–gene–disease enrichment analysis (Fig. 2F and G; Table S3) revealed strong associations with amino acid metabolism and central carbon metabolism. Key pathways included alanine, aspartate, and glutamate metabolism, cysteine and methionine metabolism, arginine and proline metabolism, glycine, serine, and threonine metabolism, and pyruvate metabolism. Glutathione metabolism further indicated impaired redox homeostasis.

In addition, pathways related to glycolysis, TCA cycle, and broader carbon metabolism were enriched, suggesting disruption of core energy-producing processes. Neuroactive ligand–receptor interaction was also enriched, indicating potential systemic signaling effects.

Metabolite–metabolite interaction network analysis (Fig. 2H and I; Table S4) further supported these findings. Highly enriched pathways included glycosaminoglycan biosynthesis, butanoate metabolism, and glyoxylate and dicarboxylate metabolism. Additional pathways such as amino sugar and nucleotide sugar metabolism, glycerolipid metabolism, and fatty acid biosynthesis indicated disruption of glycosylation and lipid metabolism. Moderately enriched pathways included oxidative phosphorylation, carbon metabolism, nitrogen metabolism, and pantothenate and CoA biosynthesis, suggesting impaired mitochondrial and metabolic function.

Network analysis of upregulated metabolites (Fig. 3C–F; Tables S4) suggested associations with metabolic adaptation and stress-response pathways. Metabolite–gene–disease enrichment (Tables S3) indicated involvement of cofactor and vitamin-related pathways, including pantothenate and CoA biosynthesis, biotin metabolism, and selenocompound metabolism, suggesting adjustments in cofactor availability and redox balance. Additional pathways related to glycolysis, TCA cycle, and amino acid metabolism were also identified, although with lower enrichment compared to downregulated metabolites. Enrichment of aminoacyl-tRNA biosynthesis further suggests potential changes in translational capacity. Metabolite–metabolite interaction analysis revealed modest enrichment in pathways related to glyoxylate metabolism and cofactor-dependent processes. Vitamin-related pathways, including those associated with flavin and B-vitamin metabolism, were also identified. Overall, these network-level changes in upregulated metabolites were more limited and likely represent compensatory responses compared to the extensive disruptions observed in downregulated metabolites.

### LC-MS profiling revealed global metabolic alterations in ischemic VSCs

**Fig. 4:**
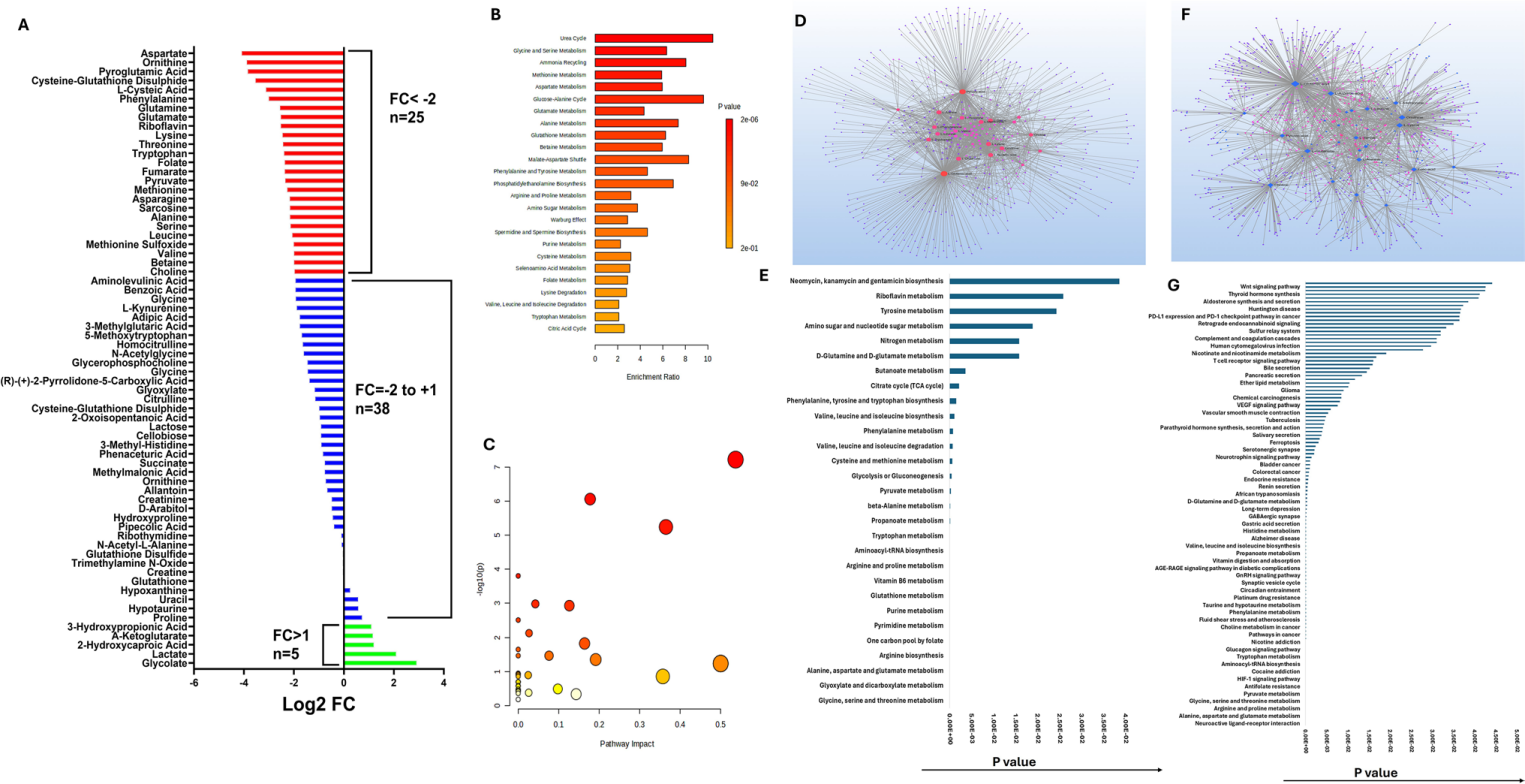
**A** Bar diagram showing 68 metabolites detected in the ischemic VSCs based on the Log2 FC. **B** MetaboAnalyst 6.0 pathway enrichment analysis of metabolites significantly downregulated (FC < 2) in ischemia challenged VSCs (*p* < 0.05). **C **MSEA using MetaboAnalyst 6.0 showing significantly impacted pathways on -log10(p) associated with the metabolites in VSCs (FC < 2) (data in Table S5a). **D** Metabolite-gene-disease interaction network and **F** metabolite-metabolite interaction network associated with downregulated metabolites in VSCs. The pathways enriched based on **E** metabolite-gene-disease interaction network and **G** metabolite-metabolite interaction network

**Fig. 5:**
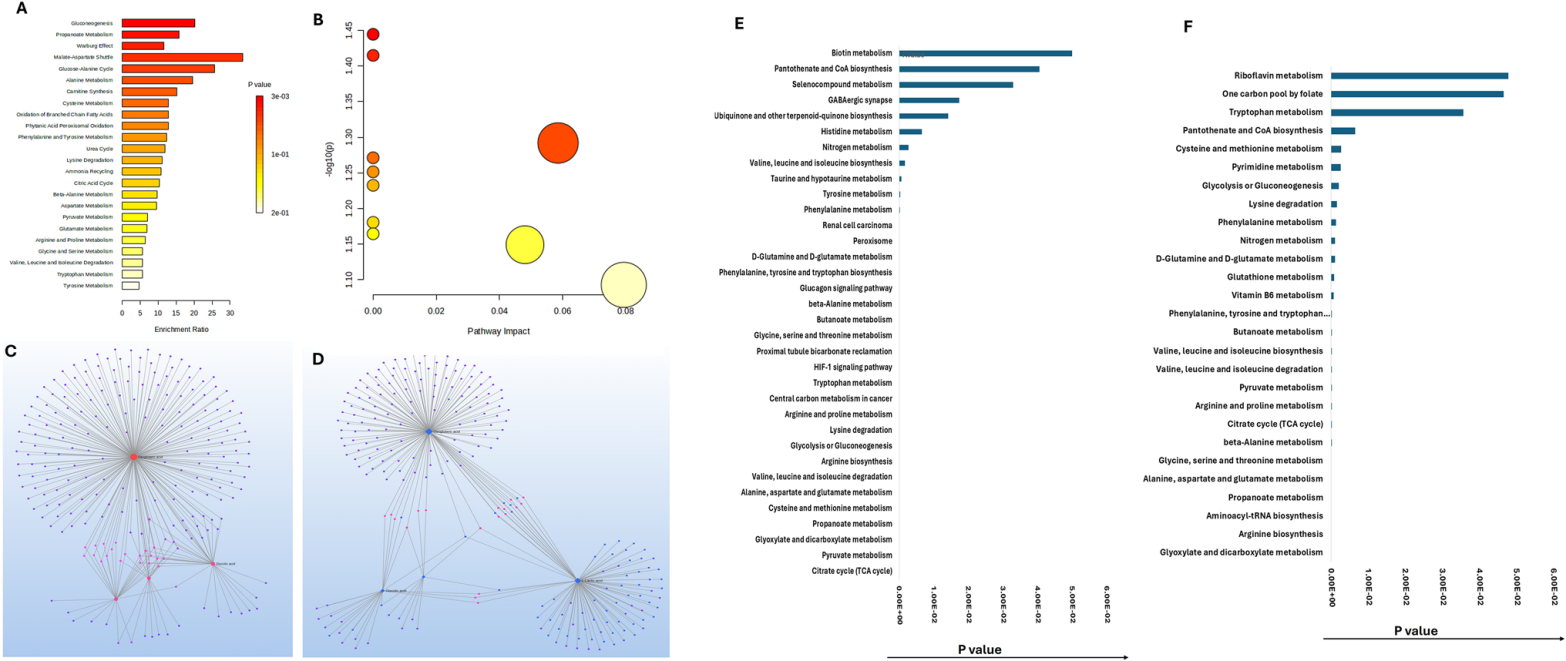
**A** MetaboAnalyst 6.0 pathway enrichment analysis of metabolites significantly upregulated in ischemia challenged VSCs (*p* < 0.05). **B** MSEA using MetaboAnalyst 6.0 showing significantly impacted pathways on -log10(p) associated with the upregulated metabolites in VSCs (data in Table S5b). **C** Metabolite-gene-disease interaction network and **D** metabolite-metabolite interaction network associated with upregulated metabolites in VSCs. The pathways enriched based on **E** metabolite-gene-disease interaction network and **F** metabolite-metabolite interaction network

Metabolomic profiling using LC-MS revealed substantial metabolomic alterations in VSCs subjected to ischemic conditions. A total of 68 metabolites were detected, of which five were upregulated, while 25 metabolites were significantly downregulated (Log2 FC < − 2.00) (Figs. 4A and 5A; Table S1b). The Log2 FC of downregulated metabolites ranged from − 2.01 to − 4.12, indicating marked attenuation of key biochemical pathways.

Amino acid metabolism was strongly affected, with significant reductions observed in branched-chain amino acids, suggesting disruptions in protein synthesis, nitrogen balance, and transamination processes critical for VSC function. Mitochondrial dysfunction was evidenced by a decrease in tricarboxylic acid (TCA) cycle intermediates, notably fumarate and pyruvate, as well as sulfur metabolism intermediate L-cysteic acid. Riboflavin, a key mitochondrial cofactor, was also downregulated. Redox imbalance was further supported by significant reductions in cysteine-glutathione disulfide and pyroglutamic acid, indicating impaired glutathione homeostasis. Ornithine, integral to the urea cycle and polyamine biosynthesis, was also markedly decreased.

In contrast, a subset of metabolites was upregulated, indicating adaptive metabolic reprogramming. Glycolate exhibited the greatest increase, followed by lactate, consistent with a glycolytic shift. Modest elevations were observed in 2-hydroxycaproic acid, α-ketoglutarate, and 3-hydroxypropionic acid, suggestive of changes in fatty acid metabolism, TCA cycle dynamics, and short-chain fatty acid turnover.

### Metabolic and Pathway alterations in ischemic VSCs

Pathway enrichment analysis of downregulated metabolites highlighted a stratified disruption across amino acid and mitochondrial metabolism (Fig. 4B and C; Table S5). The most significantly enriched pathway was alanine, aspartate, and glutamate metabolism (− log10(p) = 7.22), underscoring impaired nitrogen cycling and TCA flux. Arginine biosynthesis (− log10(p) = 6.06) and glycine, serine, and threonine metabolism (− log10(p) = 5.24) were also strongly enriched. Additional significantly enriched pathways included valine, leucine, and isoleucine biosynthesis (− log10(p) = 3.81), glyoxylate and dicarboxylate metabolism (− log10(p) = 2.98), and cysteine and methionine metabolism (− log10(p) = 2.93), reflecting impaired energy production and redox capacity. Collectively, these findings indicate a coordinated collapse of amino acid metabolism and mitochondrial function in ischemic VSCs.

Moderately enriched pathways included nitrogen metabolism (− log10(p) = 2.51), glutathione metabolism (− log10(p) = 2.12), arginine and proline metabolism (− log10(p) = 1.82), and phenylalanine metabolism (− log10(p) ≈ 1.6), suggesting broader perturbation of amino acid turnover and redox balance. Non-significant yet biologically relevant pathways included histidine metabolism (− log10(p) = 1.64), pantothenate and CoA biosynthesis, TCA cycle (− log10(p) = 1.46), and tryptophan metabolism, indicating partial preservation of mitochondrial and amino acid metabolic functions. Pathways such as purine metabolism (− log10(p) = 0.18) suggested relative preservation of nucleotide salvage pathways. Enrichment ratios further revealed strong overrepresentation in the urea cycle, glycine and serine metabolism, ammonia recycling, and glutathione metabolism, consistent with disruption of nitrogen handling and redox homeostasis (Fig. 4B and C; Table S2 and S5).

Upregulated metabolites in ischemic VSCs reflected a compensatory metabolic response. Glycolate and lactate were prominently increased, consistent with a glycolytic shift under hypoxic stress. Pathway enrichment analysis of upregulated metabolites did not yield statistically significant pathways after multiple testing correction; however, several biologically relevant trends were observed (Fig. 5A-C; Table S2 and S5).

Arginine biosynthesis (− log10(p) = 1.44), butanoate metabolism (− log10(p) = 1.41), and the TCA cycle (− log10(p) = 1.29) were among the most enriched pathways. Additional enrichment was observed in β-alanine metabolism (− log10(p) = 1.27), propanoate metabolism (− log10(p) = 1.25), and pyruvate metabolism (− log10(p) = 1.23).

Moderate enrichment of glycolysis/gluconeogenesis and pathways related to the Warburg effect further supports a shift toward glycolytic energy production, although these changes did not reach statistical significance.

### Network analyses reveal systemic metabolic and signaling collapse in ischemic VSCs

Network-based metabolite–gene–disease interaction analysis revealed a broad suppression of signaling and metabolic pathways associated with VSC proliferation, differentiation, and survival (Fig. 4D-G; Table S6 and S7).

Enriched signaling pathways included taste transduction, Wnt signaling, and Ras signaling, suggesting interference with proliferation and differentiation mechanisms. Pathways associated with cardiovascular and neurodegenerative diseases, including adrenergic signaling, Huntington disease, and PD-1/PD-L1 signaling, were also implicated. Redox and metabolic signaling disruptions were evident through alterations in endocannabinoid signaling, estrogen signaling, sulfur relay systems, complement cascades, and EGFR-related pathways.

Core metabolic pathways including HIF-1 signaling (*P* = 1.03E − 13), glycolysis/gluconeogenesis (*P* = 5.13E − 07), TCA cycle (*P* = 8.84E − 09), and pyruvate metabolism (*P* = 1.34E − 14) were significantly enriched, reflecting mitochondrial collapse and impaired energy metabolism. Strong enrichment of alanine, aspartate, and glutamate metabolism (*P* = 2.74E − 39), glycine, serine, and threonine metabolism (*P* = 9.06E − 19), and cysteine and methionine metabolism (*P* = 3.75E − 30) further confirmed widespread metabolic exhaustion across amino acid pathways.

Metabolite–metabolite interaction network analysis (Fig. 4F and G; Table S7) further demonstrated significant reductions in central metabolic pathways. The most affected pathways included glycine, serine, and threonine metabolism, glyoxylate and dicarboxylate metabolism, and alanine, aspartate, and glutamate metabolism. Additional pathways involved in amino acid biosynthesis, nucleotide metabolism, and redox regulation were also suppressed, including arginine biosynthesis, one-carbon metabolism, and glutathione metabolism.

Network-level analysis of upregulated metabolites demonstrated features of metabolic plasticity in response to ischemia (Fig. 5C–F; Table S7). Metabolite–gene–disease associations indicated enrichment in cofactor and vitamin-related pathways, including biotin metabolism, pantothenate and CoA biosynthesis, selenocompound metabolism, and ubiquinone biosynthesis, suggesting increased cofactor demand and redox regulation. Pathways related to glycolysis (*P* = 7.61E − 11), pyruvate metabolism (*P* = 4.76E − 26), and TCA cycle (*P* = 2.35E − 29) were enriched, suggesting partial engagement of central energy pathways. Additional pathways related to amino acid metabolism, including taurine, tyrosine, phenylalanine, and tryptophan metabolism, were enriched, reflecting adaptive amino acid signaling. Central metabolic pathways, including glycolysis, pyruvate metabolism, and the TCA cycle, were also enriched, consistent with metabolic reprogramming under hypoxic stress. HIF-1 signaling further supported transcriptional adaptation.

Metabolite–metabolite interaction network analysis revealed strong enrichment in glyoxylate and dicarboxylate metabolism, arginine biosynthesis, and aminoacyl-tRNA biosynthesis (*P* = 2.29E − 10), vitamin B6 metabolism (*P* = 6.81E − 04), riboflavin metabolism (*P* = 4.77E − 02), indicating activation of nitrogen metabolism and translational processes. Additional enrichment was observed in propanoate metabolism, alanine, aspartate, and glutamate metabolism, glycolysis/gluconeogenesis, and TCA cycle, reflecting coordinated activation of both oxidative and glycolytic pathways. Amino acid metabolism and redox pathways, including valine, leucine, and isoleucine degradation, arginine and proline metabolism, and glutathione metabolism, were also enriched. Cofactor-related pathways such as vitamin B6 metabolism, riboflavin metabolism, and one-carbon metabolism further suggested increased biosynthetic and antioxidant demand.

### Metabolite-specific regulation of cardiac transcription factors in ischemic EATDS and VSCs

To investigate the transcriptional consequences of ischemia-induced metabolomic reprogramming, three metabolites, sarcosine, (R)(+) 2-pyrrolidone-5-carboxylic acid (pyroglutamic acid), and 3-hydroxypropionic acid were selected for further functional evaluation. The selection was based on their significant dysregulation under ischemic conditions and involvement in key metabolic pathways relevant to redox balance, mitochondrial function, and methylation dynamics. Sarcosine and pyroglutamic acid were notably downregulated in ischemic samples, while 3-hydroxypropionic acid was upregulated. These metabolites were applied exogenously to ischemia-challenged cultures of EATDS and VSCs to assess their impact on the transcriptional regulation of genes implicated in CM-regeneration. Specifically, the expression levels of six critical CM-specific transcription factors and structural regulators, GATA4, NKX2.5, TROP-I, LGALS1, TBX5, and IRX4, were quantified by RT qPCR, relative to untreated controls.

**Fig. 6:**
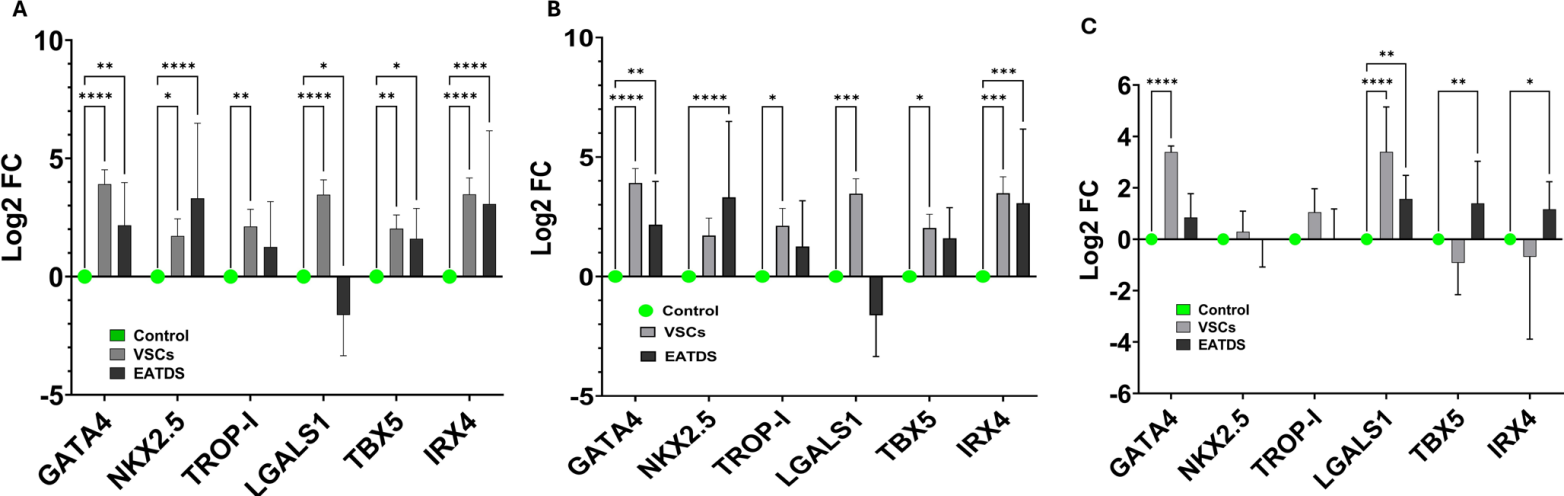
Gene expression analysis of VSCs and EATDS treated with **A** sarcosine, **B** (R)-(+)−2-Pyrrolidone-5-carboxylic acid and **C** hydroxypropionic acid showing the altered expression of the cardiac regenerative genes GATA4, NKX2.5, TROP-I, LGALS1, TBX5, and IRX4

In EATDS cells, sarcosine treatment (Fig. 6A) led to a statistically significant upregulation of GATA4 (*P* = 0.0035), NKX2.5 (*P* < 0.0001), TBX5 (*P* = 0.0369), and IRX4 (*P* < 0.0001); however, the increase in TROP-I was statistically not significant (*P* = 0.1200). Interestingly, LGALS1 displayed significant downregulation (*P* = 0.0341) compared to the control. Sarcosine-treated VSCs displayed a statistically significant upregulation of GATA4 (*P* < 0.0001), NKX2.5 (*P* = 0.0189), TROP-I (*P* = 0.0030), LGALS1 (*P* < 0.0001), TBX5 (*P* = 0.0047), and IRX4 (*P* < 0.0001) compared to the control. In EATDS cells, (R)-(+)−2-Pyrrolidone-5-carboxylic acid treatment (Fig. 6B) led to a statistically significant upregulation of GATA4 (*P* = 0.0071), NKX2.5 (*P* < 0.0001), TBX5 (*P* = 0.0561), and IRX4 (*P* < 0.0001); however, the increase in TROP-I was statistically not significant (*P* = 0.1581). Interestingly, LGALS1 displayed significant downregulation (*P* = 0.0523) compared to the control. (R)-(+)−2-Pyrrolidone-5-carboxylic acid treated VSCs displayed a statistically significant upregulation of GATA4 (*P* < 0.0001), TROP-I (*P* = 0.0229), LGALS1 (*P* < 0.0001), TBX5 (*P* = 0.0307), and IRX4 (*P* < 0.0001) compared to the control. However, the increase in Nkx2.5 was statistically not significant (*P* = 0.0766). In EATDS cells, 3-Hydroxypropionic acid treatment (Fig. 6C) led to a statistically significant upregulation of LGALS1 (*P* = 0.0016), TBX5 (*P* = 0.0054), and IRX4 (*P* = 0.0232); however, the increase in GATA4 was statistically not significant (*P* = 0.1226) compared to the control. Interestingly, the alterations in Nkx2.5 (*P* = 0.9975) and TROP-I (*P* = 0.9999) were closely similar to the control. 3-Hydroxypropionic acid treated VSCs displayed a statistically significant upregulation of GATA4 (*P* < 0.0001) and LGALS1 (*P* < 0.0001); however, the increase in Nkx2.5 (*P* = 0.8080) and TROP-I (*P* = 0.1149) were statistically not significant. TBX5 (*P* = 0.1833) and IRX4 (*P* = 0.3776) displayed downregulation; however, this was statistically not significant compared to the control. These data highlight cell-type-specific differences in transcriptional sensitivity and regulation in response to metabolite fluctuations under ischemic conditions.

## Discussion

Metabolomic profiling of ischemia-exposed EATDS and VSCs showed a significant reprogramming of metabolic networks that is consistent with hypoxic-ischemic injury. Ischemia induced a broad downregulation of intermediary metabolism, particularly amino acid biosynthesis, oxidative phosphorylation, and redox-buffering pathways, alongside partial activation of glycolytic salvage and cofactor recycling pathways. These findings align with the known metabolic consequences of myocardial ischemia, where oxygen deprivation constrains mitochondrial ATP production, promoting a metabolic shift toward anaerobic glycolysis and catabolic preservation of energy substrates (Ramachandra et al. 2020; Wal et al. 2023; Lopaschuk et al. 2021).

In EATDS, the downregulation of branched-chain amino acids, aromatic amino acids, and one-carbon metabolism intermediates (e.g., serine, glycine, folate) signifies a collapse in biosynthetic capacity and a loss of metabolic plasticity. This pattern is emblematic of ischemia-induced blockade in anabolic processes and protein turnover, with downstream effects on nucleotide synthesis, methyl-group donor availability, and redox balance (Pedley et al. 2022; Ghergurovich et al. 2020; Furuhashi 2020). The concomitant depletion of TCA cycle intermediates such as α-ketoglutarate, succinate, and fumarate underscores mitochondrial dysfunction and compromised oxidative metabolism. This is further validated by the downregulation of glutathione metabolism, indicating impaired antioxidant defense and increased vulnerability to oxidative stress and ferroptosis (Cao et al. 2019).

Additionally, pathway enrichment analysis confirmed significant disruption in nitrogen-handling pathways, including alanine, aspartate, glutamate, and glycine-serine-threonine metabolism. As these pathways are critical for transamination, ammonia detoxification, and energy integration, their decrease reflects a failure to maintain nitrogen homeostasis during ischemic stress (Kimball & Jefferson, 2001). The integration of gene-metabolite-disease association networks revealed overlapping signatures with neurodegenerative disorders such as Alzheimer’s disease and Huntington’s disease, suggesting conserved metabolic vulnerabilities across tissues with high mitochondrial and redox demands. The observed engagement of HIF-1, PI3K-Akt, and cGMP-PKG pathways reinforces the role of EATDS as an active metabolic participant in ischemic signaling and systemic remodeling (Cao et al., 2019; Spinelli & Haigis, 2018).

Interestingly, glycolate, hypoxanthine, and 3-hydroxypropionic acid were significantly upregulated in EATDS. These changes suggest an adaptive metabolic response characterized by activation of anaerobic glycolysis, short-chain fatty acid turnover, and intermediary metabolism (L. Luo & Liu, 2016; Sookoian & Pirola, 2012). Moreover, the pathway enrichment analysis related to carbohydrate metabolism (e.g., starch, galactose, and sucrose metabolism), lactate degradation, and β-alanine metabolism indicates a compensatory increase in glycolytic throughput. These patterns are consistent with HIF-1-driven transcriptional reprogramming and the Warburg effect, which has been observed in ischemic myocardium and other hypoxic tissues (Martínez et al., 2017; Baker et al., 2004; Rogatzki et al., 2015). These findings imply that EAT is not merely a structural fat depot but a dynamic metabolic tissue with the capacity for adaptive remodeling during ischemic stress via EATDS.

Similar and coordinated metabolomic reprogramming in VSCs were featured with ischemia induced robust downregulation of amino acid metabolism, mitochondrial energetics, one-carbon transfer reactions, and redox-regulating pathways. Depletion of essential and conditionally essential amino acids (e.g., methionine, glutamine, lysine, threonine), together with key intermediates in transsulfuration (e.g., cysteine, pyroglutamate), strongly indicated the suppressed biosynthesis, impaired transamination, and reduced methylation capacity (Jiang et al., 2009; Gohil et al., 2010; Jobgen et al., 2006). Decreased levels of mitochondrial cofactors and TCA intermediates, including riboflavin, fumarate, and pyruvate, further validate the collapse in bioenergetics representing mitochondrial dysfunction (Bender, 2012). Furthermore, the pathway enrichment analysis revealed that the most significantly impacted biological processes were glutathione metabolism, nitrogen metabolism, and glyoxylate dicarboxylate metabolism, which are key metabolic systems essential for cellular detoxification, maintenance of redox homeostasis, and mitochondrial function (Perry et al., 2016; Maher, 2005).

Network analysis revealed widespread dysregulation of signaling pathways governing cellular proliferation, differentiation, and stress adaptation, including Wnt, Ras, thyroid hormone, estrogen, and adrenergic pathways. Downregulation of HIF-1 signaling, glycolysis/gluconeogenesis, and pyruvate metabolism further supports the presence of a metabolically rigid VSC phenotype under ischemic stress that failed to fully activate hypoxia-responsive pathways to promote survival in energy-deprived states (Janbandhu et al. 2022). At the metabolite-metabolite network level, the significant downregulation of critical metabolic pathways including glycine, serine, and threonine metabolism; glyoxylate and dicarboxylate metabolism; arginine biosynthesis; the one-carbon folate cycle; pyrimidine synthesis; glutathione metabolism; glycolysis; pyruvate metabolism; and the TCA cycle underscores the extensive metabolic impairment in VSCs, that may affect key domains of biosynthesis, redox homeostasis, and cellular energy generation (S. Chen et al. 2023; Annibal et al. 2021; Arnold and Finley 2022; Tan et al. 2023; Bulló et al. 2021).

Despite widespread metabolic suppression, several metabolites involved in glycolysis, short-chain fatty acid turnover, and mitochondrial intermediary metabolism were found to be elevated. These include glycolate, lactate, 2-hydroxycaproic acid, α-ketoglutarate, and 3-hydroxypropionic acid, suggesting a compensatory activation of anaerobic glycolysis and alternative salvage pathways. This pattern aligns with prior evidence indicating that ischemic VSCs undergo metabolic reprogramming to maintain redox balance and energy production through glycolytic flux and intermediary substrate recycling (Mouton and Hall 2020; Z.-T. Chen et al. 2021). Gene-metabolite network analysis of these upregulated metabolites revealed enrichment in biosynthetic and redox-regulating pathways, including pantothenate and CoA biosynthesis, selenocompound metabolism, and ubiquinone biosynthesis, indicating increased demand for cofactors involved in mitochondrial repair and antioxidant capacity. These metabolic adaptations are consistent with cardiac stromal cell responses to ischemia, which involve enhanced glycolytic flux, cofactor synthesis, and antioxidant support to mitigate oxidative stress and sustain cellular viability (Liu et al. 2025a, b; Beltran et al. 2020).

To determine whether the metabolomic reprogramming observed under ischemia exerts transcriptional consequences, three metabolites, sarcosine, (R)-(+)−2-pyrrolidone-5-carboxylic acid (pyroglutamic acid), and 3-hydroxypropionic acid were selected based on their significant dysregulation and biochemical relevance. These compounds were applied exogenously to EATDS and VSC cultures, and their effects on the expression of six cardiac repair biomarkers (GATA4, NKX2.5, TROP-I, LGALS1, TBX5, IRX4) were assessed via RT-qPCR. These compounds, while not conventionally associated with cardiac biology, participate in core metabolic pathways implicated in stress responses, redox balance, and epigenetic regulation; however, they are highly relevant to ischemic injury and tissue remodeling.

Sarcosine, a methylated glycine derivative, functions as a substrate in the folate-mediated one-carbon cycle and contributes to S-adenosylmethionine-dependent methyl group transfer, which is crucial for epigenetic regulation via DNA and histone methylation (Mentch et al., 2015; Strmiska et al., 2019). Although primarily studied in the context of epithelial-mesenchymal transition and tumor progression in prostate and colon cancers (Strmiska et al., 2019) sarcosine has recently been implicated in skeletal muscle regeneration through modulation of macrophage phenotype and chromatin remodeling (Y. Liu et al. 2025a, b). In the present study, sarcosine induced robust transcriptional activation in VSCs, upregulating the CM-biomarkers, suggesting that this metabolite may potentiate VSC reprogramming through enhanced methylation activity. In EATDS, sarcosine elicited a more restricted transcriptional response, indicating cell-type-specific differences in metabolite handling or epigenetic regulation.

Pyroglutamic acid, a cyclic lactam of glutamic acid and a key intermediate in the γ-glutamyl cycle, is essential for glutathione homeostasis and redox regulation (Gamarra et al. 2019a). Accumulation of pyroglutamic acid has been observed in pathologies marked by oxidative stress, including cancers, and has been proposed as a metabolic biomarker of redox imbalance; however, its role in cardiac ischemia remain unexplored (Gamarra et al. 2019a; Stewart, 2024). Treatment with pyroglutamic acid resulted in the upregulation of cardiac regeneration markers in both EATDS and VSCs underscore its potential as a redox-sensitive modulator of transcriptional networks under ischemic stress.

3-hydroxypropionic acid, a three-carbon intermediate in propionate and β-alanine metabolism, is involved in mitochondrial energy integration and short-chain fatty acid turnover (H. Luo et al. 2016). This metabolite induced selective transcriptional upregulation of GATA4, LGALS1, TBX5, and IRX4 in EATDS and GATA4 and LGALS1 in VSCs. This targeted transcriptional profile suggests a context-dependent role, possibly linked to mitochondrial retrograde signaling or NAD+/redox sensing pathways. Given that EATDS displayed greater mitochondrial and oxidative disruption, the increased sensitivity to 3-hydroxypropionic acid could reflect differential metabolic priming or transcription factor responsiveness to mitochondrial stress signals.

Importantly, when comparing EATDS and VSCs, we observed distinct patterns of metabolic reprogramming under ischemic conditions, which suggests these stromal populations may play different roles within the cardiac microenvironment. EATDS showed more pronounced changes in one-carbon and redox-related pathways, which may indicate increased sensitivity to oxidative stress and potential alterations in epigenetic regulation. In contrast, VSCs exhibited a broader suppression of amino acid metabolism and TCA cycle intermediates, consistent with a more substantial impairment in cellular bioenergetics. These differences suggest that EATDS may retain some degree of metabolic flexibility, potentially supporting paracrine signaling, whereas VSCs may undergo a more pronounced metabolic decline that could limit their functional contribution to repair.

Overall, our findings support the hypothesis that ischemia-induced alterations in metabolite profiles contribute directly to transcriptional regulation in cardiac-associated cells especially EATDS and VSCs. The study revealed novel insights into metabolite-driven signaling that orchestrate cardiac gene expression under ischemic conditions. Despite these promising insights, the study is limited by the lack of: (1) in-depth metabolomic regulatory mechanisms, (2) combinatorial impact of metabolites in the ischemic responses, (3) in vitro models to simulate the myocardial and epicardial cross talk via metabolites, (4) protein level quantitative analysis of signature genes and downstream signaling and (5) in vivo validations. Nonetheless, the findings highlight the potential of targeting metabolomic mediators to modulate transcriptional programs involved in cardiac repair and protective remodeling. From a translational perspective, these findings suggest that targeting metabolite-associated pathways in cardiac stromal cells may represent a potential strategy to modulate post-ischemic remodeling and enhance cardiac repair, warranting further investigation in in vivo and clinical settings.

## Conclusion

This study demonstrated that ischemia altered the metabolomic content and influenced the metabolic reprogramming in EATDS and VSCs, marked by downregulation of amino acid, redox, and mitochondrial metabolism, and selective upregulation of glycolytic and stress-adaptive pathways. The key metabolites such as sarcosine, pyroglutamic acid, and 3-hydroxypropionic acid modulate transcription of cardiac regenerative genes in a cell-type specific manner. These findings suggest that ischemia-induced metabolic shifts not only compromise cellular bioenergetics but also actively influence gene expression programs accelerating the protective cardiac remodeling. Overall, such integrated metabolomic transcriptional response highlights novel therapeutic targets for modulating cellular resilience and heart regeneration following ischemic heart diseases.

## Supplementary Information

Below is the link to the electronic supplementary material.

Supplementary Material 1.

## Data Availability

Data with the raw counts matrices and assessments are available upon request from the authors through proper channels.
