# Supplementary material for: Integrated metabolomic profiling reveals metabolomic responses by epicardial and myocardial stromal cells to ischemia
Source: Metabolomics. 2026 Apr 29;22(3):61. doi: 10.1007/s11306-026-02438-0 (PMC13124829; doi:10.1007/s11306-026-02438-0)
Supplement: Supplementary file 1 — Supplementary Material 1. [file 11306_2026_2438_MOESM1_ESM.docx]

| **Supplementary Table 1.** Metabolite Abundance Changes Reported as Average Log₂ Fold-Change | | | | | | |
| --- | --- | --- | --- | --- | --- | --- |
| ***Table 1a. EAT-Derived Stromal Cells (EATDS)*** | | |  | ***Table 1b. Ventricular stromal cells (VSCs)*** | | |
| *Compound Formula* | *Compound*  *Name* | *Average value of Log2 FC* |  | *Compound Formula* | *Compound*  *Name* | *Average value of Log2 FC* |
| C2H4O3 | glycolate | 1.75 |  | C2H4O3 | glycolate | 2.94 |
| C3H6O3 | 3-Hydroxypropionic Acid | 1.47 |  | C3H6O3 | lactate | 2.12 |
| C5H4N4O | hypoxanthine | 1.31 |  | C6H12O3 | 2-Hydroxycaproic acid | 1.23 |
| C12H22O11 | cellobiose | 1.24 |  | C5H6O5 | a-ketoglutarate | 1.19 |
| C12H22O11 | Lactose | 1.24 |  | C3H6O3 | 3-Hydroxypropionic Acid | 1.13 |
| C20H32N6O12S2 | glutathione disulfide | -0.28 |  | C5H9NO2 | proline | 0.76 |
| C4H4N2O2 | uracil | -0.32 |  | C2H7NO2S | Hypotaurine | 0.61 |
| C4H6O4 | Methylmalonic acid | -0.71 |  | C4H4N2O2 | uracil | 0.60 |
| C4H9N3O2 | creatine | -1.02 |  | C5H4N4O | hypoxanthine | 0.28 |
| C8H20NO6P | Glycerophosphocholine | -1.25 |  | C10H17N3O6S | glutathione | 0.03 |
| C6H12O3 | 2-Hydroxycaproic acid | -1.34 |  | C4H9N3O2 | creatine | -0.05 |
| C10H14N2O6 | Ribothymidine | -1.40 |  | C3H9NO | Trimethylamine N-oxide | -0.06 |
| C3H6O3 | lactate | -1.53 |  | C20H32N6O12S2 | glutathione disulfide | -0.06 |
| C10H17N3O6S | glutathione | -1.79 |  | C5H9NO3 | N-Acetyl-L-alanine | -0.12 |
| C3H7NO2 | alanine | -1.82 |  | C10H14N2O6 | Ribothymidine | -0.13 |
| C3H7NO2 | sarcosine | -1.82 |  | C6H11NO2 | Pipecolic acid | -0.43 |
| C2H2O3 | glyoxylate | -1.99 |  | C5H9NO3 | hydroxyproline | -0.48 |
| C6H11NO2 | Pipecolic acid | -2.06 |  | C5H12O5 | D-Arabitol | -0.52 |
| C5H13NO | choline | -2.07 |  | C4H7N3O | Creatinine | -0.52 |
| C5H8O3 | 2-Oxoisopentanoic acid | -2.30 |  | C4H6N4O3 | allantoin | -0.70 |
| C4H8N2O3 | asparagine | -2.32 |  | C5H12N2O2 | ornithine | -0.76 |
| C10H11NO3 | Phenaceturic acid | -2.40 |  | C4H6O4 | Methylmalonic acid | -0.80 |
| C5H11NO2 | betaine | -2.41 |  | C4H6O4 | succinate | -0.80 |
| C13H22N4O8S2 | Cysteine-glutathione disulphide | -2.42 |  | C10H11NO3 | Phenaceturic acid | -0.87 |
| C5H9NO4 | glutamate | -2.46 |  | C7H11N3O2 | 3-Methyl-Histidine | -0.94 |
| C2H7NO2S | Hypotaurine | -2.48 |  | C12H22O11 | cellobiose | -0.95 |
| C10H12N2O3 | L-Kynurenine | -2.68 |  | C12H22O11 | Lactose | -0.95 |
| C3H4O3 | pyruvate | -2.73 |  | C5H8O3 | 2-Oxoisopentanoic acid | -1.00 |
| C4H7NO3 | N-Acetylglycine | -2.80 |  | C13H22N4O8S2 | Cysteine-glutathione disulphide | -1.02 |
| C6H13N3O3 | citrulline | -2.88 |  | C6H13N3O3 | citrulline | -1.18 |
| C4H7N3O | Creatinine | -2.89 |  | C2H2O3 | glyoxylate | -1.20 |
| C3H9NO | Trimethylamine N-oxide | -2.93 |  | C5H7NO3 | (R)-(+)-2-Pyrrolidone-5-carboxylic acid | -1.41 |
| C4H6N4O3 | allantoin | -2.95 |  | C2H5NO2 | glycine | -1.48 |
| C6H13NO2 | leucine | -3.02 |  | C8H20NO6P | Glycerophosphocholine | -1.49 |
| C4H9NO3 | threonine | -3.09 |  | C4H7NO3 | N-Acetylglycine | -1.64 |
| C5H11NO2S | methionine | -3.26 |  | C7H15N3O3 | Homocitrulline | -1.68 |
| C5H9NO3 | N-Acetyl-L-alanine | -3.30 |  | C12H14N2O3 | 5-methoxytryptophan | -1.72 |
| C7H6O2 | Benzoic acid | -3.32 |  | C6H10O4 | 3-Methylglutaric acid | -1.80 |
| C2H5NO2 | glycine | -3.41 |  | C6H10O4 | Adipic acid | -1.80 |
| C7H11N3O2 | 3-Methyl-Histidine | -3.45 |  | C10H12N2O3 | L-Kynurenine | -1.92 |
| C5H12N2O2 | ornithine | -3.53 |  | C2H5NO2 | glycine | -1.96 |
| C11H12N2O2 | tryptophan | -3.56 |  | C7H6O2 | Benzoic acid | -1.97 |
| C9H11NO2 | phenylalanine | -3.67 |  | C5H9NO3 | Aminolevulinic acid | -1.97 |
| C7H15N3O3 | Homocitrulline | -3.70 |  | C5H13NO | choline | -2.01 |
| C17H20N4O6 | riboflavin | -3.75 |  | C5H11NO2 | betaine | -2.03 |
| C5H11NO3S | Methionine sulfoxide | -3.87 |  | C5H11NO2 | valine | -2.03 |
| C12H14N2O3 | 5-methoxytryptophan | -3.88 |  | C5H11NO3S | Methionine sulfoxide | -2.04 |
| C19H19N7O6 | folate | -4.05 |  | C6H13NO2 | leucine | -2.10 |
| C5H9NO2 | proline | -4.06 |  | C3H7NO3 | serine | -2.17 |
| C5H9NO3 | hydroxyproline | -4.28 |  | C3H7NO2 | alanine | -2.19 |
| C3H7NO3 | serine | -4.37 |  | C3H7NO2 | sarcosine | -2.19 |
| C3H7NO5S | L-Cysteic acid | -4.41 |  | C4H8N2O3 | asparagine | -2.20 |
| C6H10O4 | 3-Methylglutaric acid | -4.45 |  | C5H11NO2S | methionine | -2.30 |
| C6H10O4 | Adipic acid | -4.45 |  | C3H4O3 | pyruvate | -2.38 |
| C5H9NO3 | Aminolevulinic acid | -4.48 |  | C4H4O4 | fumarate | -2.40 |
| C4H7NO4 | aspartate | -4.49 |  | C19H19N7O6 | folate | -2.40 |
| C5H11NO2 | valine | -4.68 |  | C11H12N2O2 | tryptophan | -2.43 |
| C4H6O4 | succinate | -5.02 |  | C4H9NO3 | threonine | -2.47 |
| C5H7NO3 | Pyroglutamic acid | -5.42 |  | C6H14N2O2 | lysine | -2.49 |
| C2H5NO2 | glycine | -5.46 |  | C17H20N4O6 | riboflavin | -2.56 |
| C5H6O5 | a-ketoglutarate | -5.53 |  | C5H9NO4 | glutamate | -2.56 |
| C4H4O4 | fumarate | -5.84 |  | C5H10N2O3 | glutamine | -2.59 |
| C5H7NO3 | (R)-(+)-2-Pyrrolidone-5-carboxylic acid | -5.86 |  | C9H11NO2 | phenylalanine | -3.04 |
| C5H12O5 | D-Arabitol | -7.98 |  | C3H7NO5S | L-Cysteic acid | -3.15 |
| C6H14N2O2 | lysine | -8.40 |  | C13H22N4O8S2 | Cysteine-glutathione disulphide | -3.57 |
|  |  |  |  | C5H7NO3 | Pyroglutamic acid | -3.88 |
|  |  |  |  | C5H12N2O2 | ornithine | -3.92 |
|  |  |  |  | C4H7NO4 | aspartate | -4.12 |

| **Supplementary Table 2a**. Pathway enrichment statistics for Log₂ FC for EATDS (**Log₂ FC (-2 to –4))** | | | | | | | | |
| --- | --- | --- | --- | --- | --- | --- | --- | --- |
| ***Log₂ FC (–2 to –4)*** | ***Total*** | ***Expected*** | ***Hits*** | ***Raw p*** | ***-log(p)*** | ***Holm adjust*** | ***FDR*** | ***Impact*** |
| **Glycine, serine and threonine metabolism** | **34** | **0.44213** | 5 | 4.77E-05 | 4.3215 | 0.003816 | 0.003816 | 0.35695 |
| Valine, leucine and isoleucine biosynthesis | 8 | 0.10403 | 3 | 0.000101 | 3.9949 | 0.007994 | 0.004047 | 0 |
| Arginine biosynthesis | 14 | 0.18205 | 3 | 0.000626 | 3.2037 | 0.048797 | 0.016683 | 0.40609 |
| Glutathione metabolism | 28 | 0.36411 | 3 | 0.005011 | 2.3001 | 0.3858 | 0.080167 | 0.10839 |
| Alanine, aspartate and glutamate metabolism | 28 | 0.36411 | 3 | 0.005011 | 2.3001 | 0.3858 | 0.080167 | 0.19712 |
| Phenylalanine metabolism | 10 | 0.13004 | 2 | 0.006795 | 2.1678 | 0.5096 | 0.083857 | 0.35714 |
| Glyoxylate and dicarboxylate metabolism | 32 | 0.41612 | 3 | 0.007338 | 2.1345 | 0.54298 | 0.083857 | 0.10582 |
| Arginine and proline metabolism | 36 | 0.46814 | 3 | 0.010217 | 1.9907 | 0.74581 | 0.10217 | 0.16395 |
| Lipoic acid metabolism | 27 | 0.35111 | 2 | 0.046435 | 1.3332 | 1 | 0.37134 | 0.00189 |
| Phenylalanine, tyrosine and tryptophan biosynthesis | 4 | 0.052016 | 1 | 0.051059 | 1.2919 | 1 | 0.37134 | 0.5 |
| Riboflavin metabolism | 4 | 0.052016 | 1 | 0.051059 | 1.2919 | 1 | 0.37134 | 0.5 |
| Porphyrin metabolism | 31 | 0.40312 | 2 | 0.059643 | 1.2244 | 1 | 0.39762 | 0 |
| Cysteine and methionine metabolism | 33 | 0.42913 | 2 | 0.066686 | 1.176 | 1 | 0.41038 | 0.10446 |
| Nitrogen metabolism | 6 | 0.078023 | 1 | 0.07565 | 1.1212 | 1 | 0.43228 | 0 |
| Valine, leucine and isoleucine degradation | 40 | 0.52016 | 2 | 0.093348 | 1.0299 | 1 | 0.46887 | 0.01084 |
| Tryptophan metabolism | 41 | 0.53316 | 2 | 0.097385 | 1.0115 | 1 | 0.46887 | 0.23722 |
| Taurine and hypotaurine metabolism | 8 | 0.10403 | 1 | 0.099634 | 1.0016 | 1 | 0.46887 | 0.4 |
| Butanoate metabolism | 15 | 0.19506 | 1 | 0.17901 | 0.74713 | 1 | 0.7956 | 0 |
| Histidine metabolism | 16 | 0.20806 | 1 | 0.18979 | 0.72173 | 1 | 0.79912 | 0 |
| Pantothenate and CoA biosynthesis | 20 | 0.26008 | 1 | 0.23159 | 0.63529 | 1 | 0.88223 | 0 |
| Citrate cycle (TCA cycle) | 20 | 0.26008 | 1 | 0.23159 | 0.63529 | 1 | 0.88223 | 0.04634 |
| Pyruvate metabolism | 23 | 0.29909 | 1 | 0.26158 | 0.5824 | 1 | 0.95119 | 0.19137 |
| Glycolysis / Gluconeogenesis | 26 | 0.3381 | 1 | 0.29046 | 0.53692 | 1 | 1 | 0.09785 |
| Lysine degradation | 30 | 0.39012 | 1 | 0.3273 | 0.48506 | 1 | 1 | 0 |
| Glycerophospholipid metabolism | 36 | 0.46814 | 1 | 0.37917 | 0.42117 | 1 | 1 | 0.02582 |
| Tyrosine metabolism | 42 | 0.54616 | 1 | 0.42722 | 0.36935 | 1 | 1 | 0 |
| Primary bile acid biosynthesis | 46 | 0.59818 | 1 | 0.45727 | 0.33982 | 1 | 1 | 0.02239 |
| Purine metabolism | 71 | 0.92328 | 1 | 0.61376 | 0.212 | 1 | 1 | 0 |
|  |  |  |  |  |  |  |  |  |

| **Supplementary Table 2b**. Pathway enrichment statistics for Log₂ FC for EATDS (**Log₂ FC (–4 to –8))** | | | | | | | | |
| --- | --- | --- | --- | --- | --- | --- | --- | --- |
| ***Log₂ FC (–4 to –8)*** | ***Total*** | ***Expected*** | ***Hits*** | ***Raw p*** | ***-log(p)*** | ***Holm adjust*** | ***FDR*** | ***Impact*** |
| Alanine, aspartate and glutamate metabolism | 28 | 0.25488 | 4 | 7.78E-05 | 4.109 | 0.006225 | 0.006225 | 0.27404 |
| Arginine biosynthesis | 14 | 0.12744 | 3 | 0.000206 | 3.6854 | 0.0163 | 0.008253 | 0 |
| Citrate cycle (TCA cycle) | 20 | 0.18205 | 3 | 0.000626 | 3.2037 | 0.048797 | 0.016683 | 0.1211 |
| Glycine, serine and threonine metabolism | 34 | 0.30949 | 3 | 0.003044 | 2.5165 | 0.23442 | 0.060888 | 0.48837 |
| Butanoate metabolism | 15 | 0.13654 | 2 | 0.007554 | 2.1218 | 0.57411 | 0.12086 | 0 |
| Pantothenate and CoA biosynthesis | 20 | 0.18205 | 2 | 0.013318 | 1.8756 | 0.99884 | 0.17757 | 0 |
| Lipoic acid metabolism | 27 | 0.24577 | 2 | 0.023723 | 1.6248 | 1 | 0.25157 | 0.00189 |
| Glutathione metabolism | 28 | 0.25488 | 2 | 0.025416 | 1.5949 | 1 | 0.25157 | 0.09582 |
| Porphyrin metabolism | 31 | 0.28218 | 2 | 0.030782 | 1.5117 | 1 | 0.25157 | 0.02795 |
| Glyoxylate and dicarboxylate metabolism | 32 | 0.29129 | 2 | 0.032664 | 1.4859 | 1 | 0.25157 | 0.14815 |
| Cysteine and methionine metabolism | 33 | 0.30039 | 2 | 0.034591 | 1.461 | 1 | 0.25157 | 0.02184 |
| Arginine and proline metabolism | 36 | 0.3277 | 2 | 0.040636 | 1.3911 | 1 | 0.27091 | 0.03837 |
| Valine, leucine and isoleucine biosynthesis | 8 | 0.072822 | 1 | 0.070699 | 1.1506 | 1 | 0.404 | 0 |
| Taurine and hypotaurine metabolism | 8 | 0.072822 | 1 | 0.070699 | 1.1506 | 1 | 0.404 | 0 |
| One carbon pool by folate | 9 | 0.081925 | 1 | 0.079203 | 1.1013 | 1 | 0.42242 | 0 |
| Biotin metabolism | 10 | 0.091027 | 1 | 0.087634 | 1.0573 | 1 | 0.43817 | 0 |
| D-Amino acid metabolism | 15 | 0.13654 | 1 | 0.12872 | 0.89034 | 1 | 0.57211 | 0 |
| Nicotinate and nicotinamide metabolism | 15 | 0.13654 | 1 | 0.12872 | 0.89034 | 1 | 0.57211 | 0 |
| Histidine metabolism | 16 | 0.14564 | 1 | 0.13673 | 0.86412 | 1 | 0.57572 | 0 |
| beta-Alanine metabolism | 21 | 0.19116 | 1 | 0.17576 | 0.75507 | 1 | 0.69423 | 0 |
| Propanoate metabolism | 22 | 0.20026 | 1 | 0.18337 | 0.73667 | 1 | 0.69423 | 0 |
| Pyruvate metabolism | 23 | 0.20936 | 1 | 0.19091 | 0.71917 | 1 | 0.69423 | 0 |
| Folate biosynthesis | 27 | 0.24577 | 1 | 0.22044 | 0.65672 | 1 | 0.76673 | 0 |
| Lysine degradation | 30 | 0.27308 | 1 | 0.24192 | 0.61633 | 1 | 0.8064 | 0 |
| Sphingolipid metabolism | 32 | 0.29129 | 1 | 0.25593 | 0.59187 | 1 | 0.81899 | 0 |
| Valine, leucine and isoleucine degradation | 40 | 0.36411 | 1 | 0.30962 | 0.50917 | 1 | 0.95269 | 0 |
| Tyrosine metabolism | 42 | 0.38231 | 1 | 0.32247 | 0.49151 | 1 | 0.95547 | 0.02463 |
| Primary bile acid biosynthesis | 46 | 0.41873 | 1 | 0.3475 | 0.45904 | 1 | 0.99287 | 0.02239 |
| **Supplementary Table 2c**. Pathway enrichment statistics for Log₂ FC for EATDS (**Log₂ FC (0 to 2))**   \| ***Log₂ FC (0 to 2)*** \| ***Total*** \| ***Expected*** \| ***Hits*** \| ***Raw p*** \| ***-log(p)*** \| ***Holm adjust*** \| ***FDR*** \| ***Impact*** \| \| --- \| --- \| --- \| --- \| --- \| --- \| --- \| --- \| --- \| \| Starch and sucrose metabolism \| 18 \| 0.058518 \| 1 \| 0.057237 \| 1.2423 \| 1 \| 1 \| 0 \| \| beta-Alanine metabolism \| 21 \| 0.06827 \| 1 \| 0.066516 \| 1.1771 \| 1 \| 1 \| 0 \| \| Propanoate metabolism \| 22 \| 0.071521 \| 1 \| 0.069592 \| 1.1574 \| 1 \| 1 \| 0 \| \| Galactose metabolism \| 27 \| 0.087776 \| 1 \| 0.084855 \| 1.0713 \| 1 \| 1 \| 0.11032 \| \| Glyoxylate and dicarboxylate metabolism \| 32 \| 0.10403 \| 1 \| 0.099916 \| 1.0004 \| 1 \| 1 \| 0.07937 \| \| Purine metabolism \| 71 \| 0.23082 \| 1 \| 0.21072 \| 0.6763 \| 1 \| 1 \| 0.01829 \| | | | | | | | | |
|  |  |  |  |  |  |  |  |  |

| **Supplementary Table 3.** Network-Based Metabolite–Gene–Disease Pathway Enrichment Analysis in Ischemia-Challenged EAT-Derived Stromal Cells | | | | | | | |
| --- | --- | --- | --- | --- | --- | --- | --- |
| ***Pathway*** | ***Total*** | ***Expected*** | ***Hits*** | ***P Val*** | ***Topology*** | ***PVal.Z*** | ***Topo.Z*** |
| Alanine, aspartate and glutamate metabolism | 37 | 5.64 | 34 | 4.99e-25 | 4.09 | 5.97 | 3.66 |
| Cysteine and methionine metabolism | 52 | 7.93 | 41 | 1.7e-24 | 3.13 | 5.83 | 2.63 |
| Arginine and proline metabolism | 50 | 7.63 | 40 | 2.3e-24 | 2.59 | 5.8 | 2.05 |
| Neuroactive ligand-receptor interaction | 370 | 56.4 | 130 | 9.34e-23 | 0.377 | 5.37 | -0.323 |
| One carbon pool by folate | 39 | 5.95 | 33 | 8.9e-22 | 7.87 | 5.12 | 7.7 |
| Citrate cycle (TCA cycle) | 30 | 4.58 | 28 | 3.07e-21 | 2.9 | 4.98 | 2.39 |
| Glycine, serine and threonine metabolism | 41 | 6.25 | 32 | 4.05e-19 | 6.11 | 4.42 | 5.82 |
| Glyoxylate and dicarboxylate metabolism | 31 | 4.73 | 25 | 8.47e-16 | 2.13 | 3.55 | 1.56 |
| Arginine biosynthesis | 23 | 3.51 | 21 | 1.09e-15 | 3.38 | 3.52 | 2.9 |
| Folate transport and metabolism | 31 | 4.73 | 23 | 3e-13 | 0 | 2.88 | -0.727 |
| Pyruvate metabolism | 47 | 7.17 | 29 | 4.02e-13 | 3.96 | 2.84 | 3.51 |
| Tryptophan metabolism | 42 | 6.41 | 27 | 6.25e-13 | 2.69 | 2.79 | 2.16 |
| HIF-1 signaling pathway | 110 | 16.8 | 47 | 2.78e-12 | 1.75 | 2.62 | 1.15 |
| Glutathione metabolism | 59 | 9 | 32 | 3.44e-12 | 3.86 | 2.6 | 3.41 |
| Glutamatergic synapse | 116 | 17.7 | 48 | 6.68e-12 | 0.796 | 2.52 | 0.126 |
| Lipoic acid metabolism | 20 | 3.05 | 17 | 8.32e-12 | 1.35 | 2.5 | 0.715 |
| Propanoate metabolism | 32 | 4.88 | 22 | 1.21e-11 | 2.39 | 2.46 | 1.84 |
| Valine, leucine and isoleucine degradation | 48 | 7.32 | 27 | 5.59e-11 | 2.4 | 2.28 | 1.84 |
| Antifolate resistance | 30 | 4.58 | 20 | 2.52e-10 | 0.2 | 2.11 | -0.513 |
| Cocaine addiction | 49 | 7.47 | 25 | 4.51e-09 | 0.667 | 1.78 | -0.0128 |
| Amphetamine addiction | 69 | 10.5 | 30 | 1.6e-08 | 0.617 | 1.64 | -0.0664 |
| Calcium signaling pathway | 254 | 38.7 | 72 | 3.69e-08 | 0.667 | 1.54 | -0.0128 |
| Aminoacyl-tRNA biosynthesis | 66 | 10.1 | 27 | 3.81e-07 | 0.133 | 1.28 | -0.584 |
| Central carbon metabolism in cancer | 71 | 10.8 | 27 | 2.12e-06 | 0.862 | 1.08 | 0.197 |
| Folate biosynthesis | 28 | 4.27 | 15 | 2.67e-06 | 2.15 | 1.05 | 1.58 |
| Taurine and hypotaurine metabolism | 17 | 2.59 | 11 | 5.01e-06 | 4 | 0.982 | 3.56 |
| Tyrosine metabolism | 36 | 5.49 | 17 | 5.49e-06 | 2.64 | 0.972 | 2.11 |
| Glycolysis or Gluconeogenesis | 67 | 10.2 | 25 | 7.49e-06 | 1.64 | 0.936 | 1.03 |
| Phenylalanine metabolism | 16 | 2.44 | 10 | 2.16e-05 | 2.89 | 0.816 | 2.37 |
| Drug metabolism - other enzymes | 81 | 12.4 | 27 | 3.57e-05 | 1.6 | 0.758 | 0.987 |
| Nicotine addiction | 41 | 6.25 | 17 | 4.54e-05 | 0 | 0.731 | -0.727 |
| cAMP signaling pathway | 226 | 34.5 | 57 | 4.85e-05 | 0.713 | 0.723 | 0.0365 |
| AGE-RAGE signaling pathway in diabetic complications | 101 | 15.4 | 30 | 0.000152 | 1.39 | 0.593 | 0.766 |
| Glucagon signaling pathway | 107 | 16.3 | 31 | 0.000196 | 0.735 | 0.564 | 0.0601 |
| Long-term potentiation | 67 | 10.2 | 22 | 0.000238 | 1.62 | 0.542 | 1.01 |
| Purine metabolism | 128 | 19.5 | 35 | 0.000272 | 3.7 | 0.527 | 3.23 |
| Butanoate metabolism | 27 | 4.12 | 12 | 0.000278 | 2.08 | 0.525 | 1.5 |
| Peroxisome | 83 | 12.7 | 25 | 0.000418 | 0.0156 | 0.478 | -0.71 |
| Phenylalanine, tyrosine and tryptophan biosynthesis | 6 | 0.915 | 5 | 0.000428 | 1 | 0.475 | 0.344 |
| D-Amino acid metabolism | 6 | 0.915 | 5 | 0.000428 | 0.143 | 0.475 | -0.574 |
| Glycerophospholipid metabolism | 103 | 15.7 | 29 | 0.000528 | 3.8 | 0.451 | 3.34 |
| Valine, leucine and isoleucine biosynthesis | 4 | 0.61 | 4 | 0.000538 | 0 | 0.449 | -0.727 |
| African trypanosomiasis | 37 | 5.64 | 14 | 0.000654 | 0.393 | 0.427 | -0.306 |
| Histidine metabolism | 22 | 3.36 | 10 | 0.000727 | 1.33 | 0.415 | 0.702 |
| Synaptic vesicle cycle | 79 | 12 | 23 | 0.00118 | 0.179 | 0.36 | -0.536 |
| beta-Alanine metabolism | 31 | 4.73 | 12 | 0.00125 | 2.64 | 0.353 | 2.1 |
| Choline metabolism in cancer | 99 | 15.1 | 27 | 0.00138 | 0.846 | 0.342 | 0.18 |
| Cobalamin transport and metabolism | 18 | 2.75 | 8 | 0.00298 | 0.2 | 0.254 | -0.513 |
| Platinum drug resistance | 75 | 11.4 | 21 | 0.00321 | 0.957 | 0.246 | 0.298 |
| Vitamin digestion and absorption | 26 | 3.97 | 10 | 0.00335 | 0 | 0.241 | -0.727 |
| Fluid shear stress and atherosclerosis | 141 | 21.5 | 34 | 0.00351 | 0.859 | 0.235 | 0.193 |
| Circadian entrainment | 97 | 14.8 | 25 | 0.00465 | 1.51 | 0.203 | 0.892 |
| Proximal tubule bicarbonate reclamation | 23 | 3.51 | 9 | 0.00466 | 0.333 | 0.203 | -0.37 |
| Lysine degradation | 63 | 9.61 | 18 | 0.00486 | 1.27 | 0.198 | 0.633 |
| Bladder cancer | 41 | 6.25 | 13 | 0.00613 | 0.419 | 0.172 | -0.278 |
| Phosphonate and phosphinate metabolism | 6 | 0.915 | 4 | 0.00623 | 1 | 0.17 | 0.344 |
| Vitamin B6 metabolism | 6 | 0.915 | 4 | 0.00623 | 2.82 | 0.17 | 2.29 |
| Pathways in cancer | 533 | 81.3 | 102 | 0.00641 | 0.791 | 0.167 | 0.121 |
| Nicotinate and nicotinamide metabolism | 38 | 5.8 | 12 | 0.00862 | 3.42 | 0.133 | 2.94 |
| Diabetic cardiomyopathy | 205 | 31.3 | 44 | 0.00989 | 0.458 | 0.117 | -0.237 |
| GnRH signaling pathway | 93 | 14.2 | 23 | 0.0109 | 0.8 | 0.106 | 0.13 |
| Cholinergic synapse | 116 | 17.7 | 27 | 0.014 | 0.59 | 0.0776 | -0.0952 |
| Malaria | 50 | 7.63 | 14 | 0.0147 | 0.0789 | 0.0717 | -0.642 |
| Phospholipase D signaling pathway | 149 | 22.7 | 33 | 0.015 | 0.727 | 0.0696 | 0.0522 |
| Renin-angiotensin system | 23 | 3.51 | 8 | 0.0165 | 1.25 | 0.0587 | 0.612 |
| Porphyrin metabolism | 46 | 7.02 | 13 | 0.0171 | 1.61 | 0.0551 | 0.997 |
| Apoptosis - multiple species | 32 | 4.88 | 10 | 0.0172 | 0.657 | 0.0543 | -0.023 |
| Amoebiasis | 103 | 15.7 | 24 | 0.0195 | 0.244 | 0.0397 | -0.465 |
| Ferroptosis | 42 | 6.41 | 12 | 0.0197 | 0.212 | 0.0386 | -0.5 |
| Riboflavin metabolism | 8 | 1.22 | 4 | 0.0225 | 1.75 | 0.0234 | 1.15 |
| Inflammatory mediator regulation of TRP channels | 99 | 15.1 | 23 | 0.0227 | 0.562 | 0.0226 | -0.124 |
| Drug metabolism - cytochrome P450 | 73 | 11.1 | 18 | 0.0233 | 0.517 | 0.0194 | -0.173 |
| Dopaminergic synapse | 132 | 20.1 | 29 | 0.024 | 0.745 | 0.016 | 0.0708 |
| Renin secretion | 69 | 10.5 | 17 | 0.0273 | 0.471 | 0.00171 | -0.223 |
| Insulin secretion | 86 | 13.1 | 20 | 0.0318 | 0.395 | -0.0159 | -0.304 |
| Relaxin signaling pathway | 130 | 19.8 | 28 | 0.0334 | 0.75 | -0.0213 | 0.0765 |
| Selenocompound metabolism | 17 | 2.59 | 6 | 0.0341 | 1.07 | -0.0238 | 0.421 |
| Nitrogen metabolism | 17 | 2.59 | 6 | 0.0341 | 2 | -0.0238 | 1.42 |
| Gastric acid secretion | 76 | 11.6 | 18 | 0.0341 | 0.483 | -0.024 | -0.21 |
| Glioma | 76 | 11.6 | 18 | 0.0341 | 0.825 | -0.024 | 0.157 |
| Chagas disease | 103 | 15.7 | 23 | 0.0349 | 0.723 | -0.0263 | 0.0477 |
| Pentose phosphate pathway | 31 | 4.73 | 9 | 0.0371 | 2.23 | -0.0333 | 1.66 |
| Endocrine resistance | 99 | 15.1 | 22 | 0.0404 | 1.34 | -0.0431 | 0.708 |
| Oxytocin signaling pathway | 155 | 23.6 | 32 | 0.0415 | 1 | -0.0463 | 0.344 |
| Galactose metabolism | 32 | 4.88 | 9 | 0.0448 | 2.61 | -0.0551 | 2.07 |
| Vascular smooth muscle contraction | 134 | 20.4 | 28 | 0.0472 | 0.654 | -0.0609 | -0.0265 |
| Caffeine metabolism | 6 | 0.915 | 3 | 0.0494 | 2.12 | -0.066 | 1.55 |
| Pyrimidine metabolism | 58 | 8.85 | 14 | 0.0498 | 1.67 | -0.0671 | 1.06 |

| **Supplementary Table 4.** Metabolite-Metabolite Interaction Network in Ischemia-Challenged EATDS | | | | | | | |
| --- | --- | --- | --- | --- | --- | --- | --- |
| ***Pathway*** | ***Total*** | ***Expected*** | ***Hits*** | ***P Val*** | ***Topology*** | ***PVal.Z*** | ***Topo.Z*** |
| Glycosaminoglycan biosynthesis - heparan sulfate or heparin | 26 | 5.84 | 25 | 6.65E-16 | 4.12 | 4.26 | 3.91 |
| Butanoate metabolism | 33 | 7.42 | 27 | 4.08E-13 | 2.06 | 3.36 | 1.42 |
| Glyoxylate and dicarboxylate metabolism | 39 | 8.77 | 29 | 4.21E-12 | 2.47 | 3.03 | 1.92 |
| Mannose type O-glycan biosynthesis | 32 | 7.19 | 25 | 2.18E-11 | 1.68 | 2.8 | 0.956 |
| Propanoate metabolism | 28 | 6.29 | 21 | 3.66E-09 | 1.85 | 2.08 | 1.17 |
| Amino sugar and nucleotide sugar metabolism | 70 | 15.7 | 37 | 1.34E-08 | 1.75 | 1.9 | 1.05 |
| Glycerolipid metabolism | 21 | 4.72 | 16 | 2.12E-07 | 1.6 | 1.51 | 0.862 |
| Starch and sucrose metabolism | 14 | 3.15 | 12 | 8.35E-07 | 2.23 | 1.32 | 1.63 |
| Fatty acid biosynthesis | 36 | 8.09 | 21 | 2.75E-06 | 1.49 | 1.15 | 0.724 |
| Other types of O-glycan biosynthesis | 22 | 4.94 | 14 | 3.59E-05 | 1.38 | 0.787 | 0.597 |
| Glycosylphosphatidylinositol (GPI)-anchor biosynthesis | 9 | 2.02 | 8 | 4.44E-05 | 2.88 | 0.758 | 2.41 |
| Linoleic acid metabolism | 28 | 6.29 | 16 | 6.46E-05 | 1.78 | 0.705 | 1.08 |
| Steroid biosynthesis | 8 | 1.8 | 7 | 0.000179 | 2.14 | 0.562 | 1.52 |
| Lipoic acid metabolism | 48 | 10.8 | 22 | 0.000234 | 0.532 | 0.524 | -0.43 |
| Glycosaminoglycan biosynthesis - chondroitin sulfate or dermatan sulfate | 15 | 3.37 | 10 | 0.000295 | 1.43 | 0.491 | 0.655 |
| Oxidative phosphorylation | 40 | 8.99 | 19 | 0.000358 | 1.03 | 0.464 | 0.167 |
| 2-Oxocarboxylic acid metabolism | 23 | 5.17 | 13 | 0.000378 | 1.5 | 0.456 | 0.741 |
| Mucin type O-glycan biosynthesis | 23 | 5.17 | 13 | 0.000378 | 1.36 | 0.456 | 0.576 |
| Metabolic pathways | 26 | 5.84 | 14 | 0.00044 | 1.28 | 0.435 | 0.475 |
| Primary bile acid biosynthesis | 41 | 9.22 | 19 | 0.000531 | 1.08 | 0.408 | 0.227 |
| Carbon metabolism | 20 | 4.5 | 11 | 0.00148 | 1.42 | 0.264 | 0.646 |
| Arachidonic acid metabolism | 15 | 3.37 | 9 | 0.00181 | 0.714 | 0.236 | -0.209 |
| Nitrogen metabolism | 8 | 1.8 | 6 | 0.0023 | 0.857 | 0.203 | -0.0365 |
| Steroid hormone biosynthesis | 4 | 0.899 | 4 | 0.00252 | 2.67 | 0.189 | 2.15 |
| Inositol phosphate metabolism | 33 | 7.42 | 15 | 0.00263 | 1.16 | 0.183 | 0.325 |
| Fatty acid metabolism | 19 | 4.27 | 10 | 0.00376 | 1 | 0.133 | 0.136 |
| Glycosphingolipid biosynthesis - globo and isoglobo series | 20 | 4.5 | 9 | 0.0206 | 0.789 | -0.106 | -0.118 |
| Pantothenate and CoA biosynthesis | 6 | 1.35 | 4 | 0.0256 | 0.8 | -0.137 | -0.106 |
| Fatty acid degradation | 42 | 9.44 | 15 | 0.0336 | 0.951 | -0.175 | 0.0774 |
| Glycosaminoglycan degradation | 4 | 0.899 | 3 | 0.0376 | 3 | -0.191 | 2.56 |

| **Supplementary Table 5a**. Pathway enrichment statistics for Log₂ FC in VSCs for (**Log₂ FC (–2 to –4))** | | | | | | | | |
| --- | --- | --- | --- | --- | --- | --- | --- | --- |
| ***Log₂ FC (–2 to –4)*** | ***Total*** | ***Expected*** | ***Hits*** | ***Raw p*** | ***-log(p)*** | ***Holm adjust*** | ***FDR*** | ***Impact*** |
| Alanine, aspartate and glutamate metabolism | 28 | 0.41873 | 7 | 6.00E-08 | 7.2217 | 4.80E-06 | 4.80E-06 | 0.53686 |
| Arginine biosynthesis | 14 | 0.20936 | 5 | 8.65E-07 | 6.0628 | 6.84E-05 | 3.46E-05 | 0.17766 |
| Glycine, serine and threonine metabolism | 34 | 0.50845 | 6 | 5.70E-06 | 5.2442 | 0.000445 | 0.000152 | 0.36478 |
| Valine, leucine and isoleucine biosynthesis | 8 | 0.11964 | 3 | 0.000156 | 3.8068 | 0.012015 | 0.003121 | 0 |
| Glyoxylate and dicarboxylate metabolism | 32 | 0.47854 | 4 | 0.001037 | 2.9842 | 0.078818 | 0.015579 | 0.04233 |
| Cysteine and methionine metabolism | 33 | 0.4935 | 4 | 0.001168 | 2.9324 | 0.08763 | 0.015579 | 0.1263 |
| Nitrogen metabolism | 6 | 0.089727 | 2 | 0.003095 | 2.5093 | 0.22906 | 0.035376 | 0 |
| Glutathione metabolism | 28 | 0.41873 | 3 | 0.007504 | 2.1247 | 0.54777 | 0.075037 | 0.02675 |
| Arginine and proline metabolism | 36 | 0.53836 | 3 | 0.015125 | 1.8203 | 1 | 0.13444 | 0.16395 |
| Histidine metabolism | 16 | 0.23927 | 2 | 0.022609 | 1.6457 | 1 | 0.18087 | 0 |
| Pantothenate and CoA biosynthesis | 20 | 0.29909 | 2 | 0.034524 | 1.4619 | 1 | 0.23016 | 0 |
| Citrate cycle (TCA cycle) | 20 | 0.29909 | 2 | 0.034524 | 1.4619 | 1 | 0.23016 | 0.07615 |
| Pyruvate metabolism | 23 | 0.34395 | 2 | 0.044742 | 1.3493 | 1 | 0.27533 | 0.19137 |
| Phenylalanine, tyrosine and tryptophan biosynthesis | 4 | 0.059818 | 1 | 0.058545 | 1.2325 | 1 | 0.31224 | 0.5 |
| Riboflavin metabolism | 4 | 0.059818 | 1 | 0.058545 | 1.2325 | 1 | 0.31224 | 0.5 |
| Taurine and hypotaurine metabolism | 8 | 0.11964 | 1 | 0.1138 | 0.94384 | 1 | 0.53431 | 0 |
| Valine, leucine and isoleucine degradation | 40 | 0.59818 | 2 | 0.11844 | 0.92649 | 1 | 0.53431 | 0 |
| One carbon pool by folate | 9 | 0.13459 | 1 | 0.12713 | 0.89577 | 1 | 0.53431 | 0 |
| Tyrosine metabolism | 42 | 0.62809 | 2 | 0.12844 | 0.8913 | 1 | 0.53431 | 0.02463 |
| Biotin metabolism | 10 | 0.14954 | 1 | 0.14026 | 0.85308 | 1 | 0.53431 | 0 |
| Phenylalanine metabolism | 10 | 0.14954 | 1 | 0.14026 | 0.85308 | 1 | 0.53431 | 0.35714 |
| D-Amino acid metabolism | 15 | 0.22432 | 1 | 0.20312 | 0.69224 | 1 | 0.67708 | 0 |
| Butanoate metabolism | 15 | 0.22432 | 1 | 0.20312 | 0.69224 | 1 | 0.67708 | 0 |
| Nicotinate and nicotinamide metabolism | 15 | 0.22432 | 1 | 0.20312 | 0.69224 | 1 | 0.67708 | 0 |
| Selenocompound metabolism | 20 | 0.29909 | 1 | 0.26158 | 0.5824 | 1 | 0.83705 | 0 |
| beta-Alanine metabolism | 21 | 0.31404 | 1 | 0.27277 | 0.56421 | 1 | 0.83928 | 0 |
| Glycolysis / Gluconeogenesis | 26 | 0.38882 | 1 | 0.32632 | 0.48636 | 1 | 0.92846 | 0.09785 |
| Lipoic acid metabolism | 27 | 0.40377 | 1 | 0.33657 | 0.47293 | 1 | 0.92846 | 0 |
| Folate biosynthesis | 27 | 0.40377 | 1 | 0.33657 | 0.47293 | 1 | 0.92846 | 0 |
| Lysine degradation | 30 | 0.44863 | 1 | 0.36642 | 0.43602 | 1 | 0.96402 | 0 |
| Porphyrin metabolism | 31 | 0.46359 | 1 | 0.37609 | 0.42471 | 1 | 0.96402 | 0 |
| Sphingolipid metabolism | 32 | 0.47854 | 1 | 0.38561 | 0.41385 | 1 | 0.96402 | 0 |
| Glycerophospholipid metabolism | 36 | 0.53836 | 1 | 0.42233 | 0.37435 | 1 | 1 | 0.02582 |
| Pyrimidine metabolism | 38 | 0.56827 | 1 | 0.43989 | 0.35666 | 1 | 1 | 0 |
| Tryptophan metabolism | 41 | 0.61313 | 1 | 0.46528 | 0.33229 | 1 | 1 | 0.14305 |
| Purine metabolism | 71 | 1.0618 | 1 | 0.66549 | 0.17686 | 1 | 1 | 0 |
| **Supplementary Table 5b**. Pathway enrichment statistics for Log₂ FC in VSCs for (**Log₂ FC (0 to 2))** | | | | | | | | |
|  |  |  |  |  |  |  |  |  |
| ***Log₂ FC (0 to 2)*** | ***Total*** | ***Expected*** | ***Hits*** | ***Raw p*** | ***-log(p)*** | ***Holm adjust*** | ***FDR*** | ***Impact*** |
| Arginine biosynthesis | 14 | 0.036411 | 1 | 0.035951 | 1.4443 | 1 | 0.63044 | 0 |
| Butanoate metabolism | 15 | 0.039012 | 1 | 0.038482 | 1.4147 | 1 | 0.63044 | 0 |
| Citrate cycle (TCA cycle) | 20 | 0.052016 | 1 | 0.051059 | 1.2919 | 1 | 0.63044 | 0.05856 |
| beta-Alanine metabolism | 21 | 0.054616 | 1 | 0.053559 | 1.2712 | 1 | 0.63044 | 0 |
| Propanoate metabolism | 22 | 0.057217 | 1 | 0.056055 | 1.2514 | 1 | 0.63044 | 0 |
| Pyruvate metabolism | 23 | 0.059818 | 1 | 0.058545 | 1.2325 | 1 | 0.63044 | 0 |
| Glycolysis / Gluconeogenesis | 26 | 0.06762 | 1 | 0.065988 | 1.1805 | 1 | 0.63044 | 0 |
| Lipoic acid metabolism | 27 | 0.070221 | 1 | 0.068459 | 1.1646 | 1 | 0.63044 | 0 |
| Alanine, aspartate and glutamate metabolism | 28 | 0.072822 | 1 | 0.070925 | 1.1492 | 1 | 0.63044 | 0.04808 |
| Glyoxylate and dicarboxylate metabolism | 32 | 0.083225 | 1 | 0.08074 | 1.0929 | 1 | 0.64592 | 0.07937 |
| Arginine biosynthesis | 14 | 0.036411 | 1 | 0.035951 | 1.4443 | 1 | 0.63044 | 0 |
| Butanoate metabolism | 15 | 0.039012 | 1 | 0.038482 | 1.4147 | 1 | 0.63044 | 0 |
| Citrate cycle (TCA cycle) | 20 | 0.052016 | 1 | 0.051059 | 1.2919 | 1 | 0.63044 | 0.05856 |
| beta-Alanine metabolism | 21 | 0.054616 | 1 | 0.053559 | 1.2712 | 1 | 0.63044 | 0 |
| Propanoate metabolism | 22 | 0.057217 | 1 | 0.056055 | 1.2514 | 1 | 0.63044 | 0 |
| Pyruvate metabolism | 23 | 0.059818 | 1 | 0.058545 | 1.2325 | 1 | 0.63044 | 0 |
| Glycolysis / Gluconeogenesis | 26 | 0.06762 | 1 | 0.065988 | 1.1805 | 1 | 0.63044 | 0 |
| Lipoic acid metabolism | 27 | 0.070221 | 1 | 0.068459 | 1.1646 | 1 | 0.63044 | 0 |
| Alanine, aspartate and glutamate metabolism | 28 | 0.072822 | 1 | 0.070925 | 1.1492 | 1 | 0.63044 | 0.04808 |
| Glyoxylate and dicarboxylate metabolism | 32 | 0.083225 | 1 | 0.08074 | 1.0929 | 1 | 0.64592 | 0.07937 |

| **Supplementary Table 6.** Network-Based Metabolite–Gene–Disease Pathway Enrichment Analysis in Ischemia-Challenged VSCs | | | | | | | |
| --- | --- | --- | --- | --- | --- | --- | --- |
| ***Pathway*** | ***Total*** | ***Expected*** | ***Hits*** | ***Pval*** | ***Topology*** | ***PVal.Z*** | ***Topo.Z*** |
| Glutathione metabolism | 59 | 10.4 | 46 | 2.93E-24 | 4.04 | 5.82 | 3.43 |
| Alanine, aspartate and glutamate metabolism | 37 | 6.49 | 34 | 5.85E-23 | 4.09 | 5.47 | 3.49 |
| Cysteine and methionine metabolism | 52 | 9.12 | 41 | 4.46E-22 | 3.13 | 5.24 | 2.49 |
| Arginine and proline metabolism | 50 | 8.77 | 40 | 5.35E-22 | 2.59 | 5.22 | 1.93 |
| One carbon pool by folate | 39 | 6.84 | 33 | 8.34E-20 | 7.87 | 4.64 | 7.4 |
| Citrate cycle (TCA cycle) | 30 | 5.26 | 28 | 1.55E-19 | 2.9 | 4.57 | 2.25 |
| Neuroactive ligand-receptor interaction | 370 | 64.9 | 133 | 1.54E-18 | 0.384 | 4.3 | -0.362 |
| Glycine, serine and threonine metabolism | 41 | 7.19 | 33 | 1.70E-18 | 6.17 | 4.29 | 5.64 |
| Glyoxylate and dicarboxylate metabolism | 31 | 5.44 | 27 | 4.55E-17 | 2.3 | 3.91 | 1.63 |
| Arginine biosynthesis | 23 | 4.04 | 21 | 2.01E-14 | 3.38 | 3.21 | 2.75 |
| Folate transport and metabolism | 31 | 5.44 | 25 | 2.51E-14 | 0 | 3.19 | -0.76 |
| Drug metabolism - other enzymes | 81 | 14.2 | 44 | 5.13E-14 | 2.55 | 3.1 | 1.89 |
| Propanoate metabolism | 32 | 5.61 | 24 | 1.45E-12 | 2.57 | 2.72 | 1.9 |
| Pyruvate metabolism | 47 | 8.25 | 30 | 1.90E-12 | 4.04 | 2.69 | 3.43 |
| Antifolate resistance | 30 | 5.26 | 23 | 1.96E-12 | 0.24 | 2.68 | -0.511 |
| Tryptophan metabolism | 42 | 7.37 | 28 | 2.15E-12 | 2.69 | 2.67 | 2.03 |
| Glutamatergic synapse | 116 | 20.4 | 51 | 2.14E-11 | 0.837 | 2.41 | 0.108 |
| Lipoic acid metabolism | 20 | 3.51 | 17 | 8.50E-11 | 1.35 | 2.25 | 0.637 |
| HIF-1 signaling pathway | 110 | 19.3 | 48 | 1.16E-10 | 2.22 | 2.21 | 1.54 |
| Valine, leucine and isoleucine degradation | 48 | 8.42 | 28 | 2.30E-10 | 2.46 | 2.14 | 1.79 |
| Amphetamine addiction | 69 | 12.1 | 32 | 2.56E-08 | 0.617 | 1.59 | -0.12 |
| Cocaine addiction | 49 | 8.6 | 25 | 8.38E-08 | 0.667 | 1.46 | -0.0682 |
| Folate biosynthesis | 28 | 4.91 | 17 | 3.86E-07 | 2.3 | 1.28 | 1.63 |
| Aminoacyl-tRNA biosynthesis | 66 | 11.6 | 29 | 4.87E-07 | 0.133 | 1.25 | -0.621 |
| Calcium signaling pathway | 254 | 44.6 | 76 | 5.31E-07 | 0.667 | 1.24 | -0.0682 |
| Pyrimidine metabolism | 58 | 10.2 | 25 | 4.59E-06 | 2.94 | 0.997 | 2.29 |
| Tyrosine metabolism | 36 | 6.32 | 18 | 8.09E-06 | 2.76 | 0.931 | 2.1 |
| Platinum drug resistance | 75 | 13.2 | 29 | 1.10E-05 | 0.957 | 0.896 | 0.233 |
| Glucagon signaling pathway | 107 | 18.8 | 37 | 1.47E-05 | 0.837 | 0.862 | 0.108 |
| Fluid shear stress and atherosclerosis | 141 | 24.7 | 45 | 1.96E-05 | 0.989 | 0.829 | 0.266 |
| Taurine and hypotaurine metabolism | 17 | 2.98 | 11 | 2.04E-05 | 4 | 0.825 | 3.39 |
| Glycolysis or Gluconeogenesis | 67 | 11.8 | 26 | 2.89E-05 | 1.7 | 0.785 | 1 |
| Central carbon metabolism in cancer | 71 | 12.5 | 27 | 3.12E-05 | 0.862 | 0.776 | 0.135 |
| Cobalamin transport and metabolism | 18 | 3.16 | 11 | 4.41E-05 | 0.4 | 0.736 | -0.345 |
| Nicotine addiction | 41 | 7.19 | 18 | 7.43E-05 | 0 | 0.676 | -0.76 |
| beta-Alanine metabolism | 31 | 5.44 | 15 | 7.57E-05 | 3.36 | 0.674 | 2.73 |
| Phenylalanine metabolism | 16 | 2.81 | 10 | 7.64E-05 | 2.89 | 0.673 | 2.24 |
| Long-term potentiation | 67 | 11.8 | 25 | 8.77E-05 | 1.71 | 0.657 | 1.01 |
| AGE-RAGE signaling pathway in diabetic complications | 101 | 17.7 | 32 | 0.000353 | 1.59 | 0.497 | 0.89 |
| Drug metabolism - cytochrome P450 | 73 | 12.8 | 25 | 0.000417 | 0.517 | 0.478 | -0.224 |
| cAMP signaling pathway | 226 | 39.7 | 59 | 0.000667 | 0.736 | 0.424 | 0.00336 |
| Chemical carcinogenesis - DNA adducts | 71 | 12.5 | 24 | 0.00067 | 0.703 | 0.423 | -0.0308 |
| African trypanosomiasis | 37 | 6.49 | 15 | 0.000827 | 0.393 | 0.399 | -0.352 |
| Phenylalanine, tyrosine and tryptophan biosynthesis | 6 | 1.05 | 5 | 0.000846 | 1 | 0.396 | 0.278 |
| D-Amino acid metabolism | 6 | 1.05 | 5 | 0.000846 | 0.143 | 0.396 | -0.612 |
| Purine metabolism | 128 | 22.5 | 37 | 0.000929 | 3.86 | 0.385 | 3.24 |
| Valine, leucine and isoleucine biosynthesis | 4 | 0.702 | 4 | 0.000943 | 0 | 0.384 | -0.76 |
| Butanoate metabolism | 27 | 4.74 | 12 | 0.00104 | 2.08 | 0.372 | 1.39 |
| Pentose phosphate pathway | 31 | 5.44 | 13 | 0.00126 | 3.04 | 0.35 | 2.39 |
| Peroxisome | 83 | 14.6 | 26 | 0.00148 | 0.0156 | 0.332 | -0.744 |
| Synaptic vesicle cycle | 79 | 13.9 | 25 | 0.00154 | 0.214 | 0.327 | -0.537 |
| Histidine metabolism | 22 | 3.86 | 10 | 0.00223 | 1.33 | 0.285 | 0.623 |
| Vitamin digestion and absorption | 26 | 4.56 | 11 | 0.00272 | 0 | 0.262 | -0.76 |
| Lysine degradation | 63 | 11.1 | 20 | 0.00422 | 1.38 | 0.211 | 0.677 |
| Glycerophospholipid metabolism | 103 | 18.1 | 29 | 0.00484 | 3.8 | 0.196 | 3.18 |
| Pathways in cancer | 533 | 93.5 | 116 | 0.00518 | 0.835 | 0.188 | 0.106 |
| Choline metabolism in cancer | 99 | 17.4 | 28 | 0.0052 | 0.923 | 0.187 | 0.198 |
| Metabolism of xenobiotics by cytochrome P450 | 79 | 13.9 | 23 | 0.0074 | 1.48 | 0.147 | 0.778 |
| Bladder cancer | 41 | 7.19 | 14 | 0.00774 | 0.419 | 0.141 | -0.325 |
| Malaria | 50 | 8.77 | 16 | 0.00918 | 0.0789 | 0.122 | -0.678 |
| Phosphonate and phosphinate metabolism | 6 | 1.05 | 4 | 0.0105 | 1 | 0.107 | 0.278 |
| Vitamin B6 metabolism | 6 | 1.05 | 4 | 0.0105 | 2.82 | 0.107 | 2.16 |
| Renin-angiotensin system | 23 | 4.04 | 9 | 0.0119 | 1.25 | 0.092 | 0.537 |
| Proximal tubule bicarbonate reclamation | 23 | 4.04 | 9 | 0.0119 | 0.333 | 0.092 | -0.414 |
| Chemical carcinogenesis - reactive oxygen species | 227 | 39.8 | 53 | 0.0142 | 0.519 | 0.0713 | -0.221 |
| Base excision repair | 44 | 7.72 | 14 | 0.0151 | 0 | 0.0644 | -0.76 |
| Apoptosis - multiple species | 32 | 5.61 | 11 | 0.0165 | 0.686 | 0.0541 | -0.0484 |
| Dopaminergic synapse | 132 | 23.2 | 33 | 0.0184 | 0.851 | 0.0417 | 0.123 |
| Selenocompound metabolism | 17 | 2.98 | 7 | 0.0191 | 1.21 | 0.0372 | 0.5 |
| Porphyrin metabolism | 46 | 8.07 | 14 | 0.0224 | 1.61 | 0.019 | 0.909 |
| Ferroptosis | 42 | 7.37 | 13 | 0.0238 | 0.212 | 0.0123 | -0.54 |
| Nicotinate and nicotinamide metabolism | 38 | 6.67 | 12 | 0.0251 | 3.42 | 0.0062 | 2.79 |
| Renin secretion | 69 | 12.1 | 19 | 0.0254 | 0.471 | 0.00442 | -0.272 |
| Circadian entrainment | 97 | 17 | 25 | 0.026 | 1.51 | 0.00196 | 0.808 |
| Diabetic cardiomyopathy | 205 | 36 | 47 | 0.0274 | 0.47 | -0.00413 | -0.272 |
| GnRH signaling pathway | 93 | 16.3 | 24 | 0.0283 | 0.85 | -0.00799 | 0.122 |
| Chagas disease | 103 | 18.1 | 26 | 0.0301 | 0.815 | -0.015 | 0.0861 |
| Amoebiasis | 103 | 18.1 | 26 | 0.0301 | 0.289 | -0.015 | -0.46 |
| Thyroid hormone synthesis | 75 | 13.2 | 20 | 0.031 | 0.255 | -0.0183 | -0.495 |
| Inflammatory mediator regulation of TRP channels | 99 | 17.4 | 25 | 0.0329 | 0.625 | -0.0251 | -0.111 |
| Riboflavin metabolism | 8 | 1.4 | 4 | 0.0364 | 1.75 | -0.0367 | 1.06 |
| Galactose metabolism | 32 | 5.61 | 10 | 0.0419 | 2.74 | -0.0531 | 2.08 |
| Cholinergic synapse | 116 | 20.4 | 28 | 0.043 | 0.615 | -0.0561 | -0.121 |
| Ubiquinone and other terpenoid-quinone biosynthesis | 12 | 2.11 | 5 | 0.0442 | 0.857 | -0.0591 | 0.129 |

| **Supplementary Table 7.** Metabolite-Metabolite Interaction Network in Ischemia-Challenged VSCs | | | | | | | |
| --- | --- | --- | --- | --- | --- | --- | --- |
| ***Pathway*** | ***Total*** | ***Expected*** | ***Hits*** | ***Pval*** | ***Topology*** | ***PVal.Z*** | ***Topo.Z*** |
| Glycosaminoglycan biosynthesis - heparan sulfate or heparin | 26 | 5.84 | 25 | 6.65E-16 | 4.12 | 4.26 | 3.91 |
| Butanoate metabolism | 33 | 7.42 | 27 | 4.08E-13 | 2.06 | 3.36 | 1.42 |
| Glyoxylate and dicarboxylate metabolism | 39 | 8.77 | 29 | 4.21E-12 | 2.47 | 3.03 | 1.92 |
| Mannose type O-glycan biosynthesis | 32 | 7.19 | 25 | 2.18E-11 | 1.68 | 2.8 | 0.956 |
| Propanoate metabolism | 28 | 6.29 | 21 | 3.66E-09 | 1.85 | 2.08 | 1.17 |
| Amino sugar and nucleotide sugar metabolism | 70 | 15.7 | 37 | 1.34E-08 | 1.75 | 1.9 | 1.05 |
| Glycerolipid metabolism | 21 | 4.72 | 16 | 2.12E-07 | 1.6 | 1.51 | 0.862 |
| Starch and sucrose metabolism | 14 | 3.15 | 12 | 8.35E-07 | 2.23 | 1.32 | 1.63 |
| Fatty acid biosynthesis | 36 | 8.09 | 21 | 2.75E-06 | 1.49 | 1.15 | 0.724 |
| Other types of O-glycan biosynthesis | 22 | 4.94 | 14 | 3.59E-05 | 1.38 | 0.787 | 0.597 |
| Glycosylphosphatidylinositol (GPI)-anchor biosynthesis | 9 | 2.02 | 8 | 4.44E-05 | 2.88 | 0.758 | 2.41 |
| Linoleic acid metabolism | 28 | 6.29 | 16 | 6.46E-05 | 1.78 | 0.705 | 1.08 |
| Steroid biosynthesis | 8 | 1.8 | 7 | 0.000179 | 2.14 | 0.562 | 1.52 |
| Lipoic acid metabolism | 48 | 10.8 | 22 | 0.000234 | 0.532 | 0.524 | -0.43 |
| Glycosaminoglycan biosynthesis - chondroitin sulfate or dermatan sulfate | 15 | 3.37 | 10 | 0.000295 | 1.43 | 0.491 | 0.655 |
| Oxidative phosphorylation | 40 | 8.99 | 19 | 0.000358 | 1.03 | 0.464 | 0.167 |
| 2-Oxocarboxylic acid metabolism | 23 | 5.17 | 13 | 0.000378 | 1.5 | 0.456 | 0.741 |
| Mucin type O-glycan biosynthesis | 23 | 5.17 | 13 | 0.000378 | 1.36 | 0.456 | 0.576 |
| Metabolic pathways | 26 | 5.84 | 14 | 0.00044 | 1.28 | 0.435 | 0.475 |
| Primary bile acid biosynthesis | 41 | 9.22 | 19 | 0.000531 | 1.08 | 0.408 | 0.227 |
| Carbon metabolism | 20 | 4.5 | 11 | 0.00148 | 1.42 | 0.264 | 0.646 |
| Arachidonic acid metabolism | 15 | 3.37 | 9 | 0.00181 | 0.714 | 0.236 | -0.209 |
| Nitrogen metabolism | 8 | 1.8 | 6 | 0.0023 | 0.857 | 0.203 | -0.0365 |
| Steroid hormone biosynthesis | 4 | 0.899 | 4 | 0.00252 | 2.67 | 0.189 | 2.15 |
| Inositol phosphate metabolism | 33 | 7.42 | 15 | 0.00263 | 1.16 | 0.183 | 0.325 |
| Fatty acid metabolism | 19 | 4.27 | 10 | 0.00376 | 1 | 0.133 | 0.136 |
| Glycosphingolipid biosynthesis - globo and isoglobo series | 20 | 4.5 | 9 | 0.0206 | 0.789 | -0.106 | -0.118 |
| Pantothenate and CoA biosynthesis | 6 | 1.35 | 4 | 0.0256 | 0.8 | -0.137 | -0.106 |
| Fatty acid degradation | 42 | 9.44 | 15 | 0.0336 | 0.951 | -0.175 | 0.0774 |
| Glycosaminoglycan degradation | 4 | 0.899 | 3 | 0.0376 | 3 | -0.191 | 2.56 |
